# Molded Part Warpage Optimization Using Inverse Contouring Method

**DOI:** 10.3390/polym17172278

**Published:** 2025-08-22

**Authors:** Damir Godec, Filip Panđa, Mislav Tujmer, Katarina Monkova

**Affiliations:** 1 Faculty of Mechanical Engineering and Naval Architecture, University of Zagreb, HR-10000 Zagreb, Croatia; mislav.tujmer@fsb.unizg.hr; 2 Yazaki-Europe Limited, HR-10000 Zagreb, Croatia; 3 Faculty of Manufacturing Technologies with a seat in Prešov, Technical University Košice, 08001 Prešov, Slovakia; katarina.monkova@tuke.sk; 4 Faculty of Technology, Tomas Bata University in Zlin, Vavreckova 5669, 760 01 Zlin, Czech Republic

**Keywords:** injection molding, warpage, simulation, optimization, inverse contouring

## Abstract

Warpage is among the most prevalent defects affecting injection molded parts. In this study, we aimed to develop methods to minimize warpage through mold design. Common strategies include matching the cavity geometry to the intended shape of the part, adjusting cavity dimensions to offset material shrinkage, and optimizing the cooling system and critical injection molding parameters. These optimization methods can offer significant improvements, but recently introduced methods that optimize the molded part and mold cavity shape result in higher levels of warpage reduction. In these methods, optimization of the shape of the molded part is achieved by shaping it in the opposite direction of warpage—a method known as inverse contouring. Inverse contouring of molded parts is a design technique in which mold cavities are intentionally modified to incorporate compensatory geometric deviations in regions anticipated to exhibit significant warpage. The final result after molded part ejection and warpage is a significant reduction in deviations between the warped and reference molded part geometries. In this study, a two-step approach for minimizing warpage was used: the first step was optimizing the most significant injection molding parameters, and the second was inverse contouring. In the first step, Response Surface Methodology (RSM) and Autodesk Moldflow Insight 2023 simulations were used to optimize molded part warpage based on three processing parameters: melt temperature, target mold temperature, and coolant temperature. For improved accuracy, a Computer-Aided Design (CAD) model of the warped molded part was exported into ZEISS Inspect 2023 software and aligned with the reference CAD geometry of the molded part. The maximal warpage value after the initial simulation was 1.85 mm based on Autodesk Moldflow Insight simulations and 1.67 mm based on ZEISS Inspect alignment. After RSM optimization, the maximal warpage was 0.73 mm. In the second step, inverse contouring was performed on the molded part, utilizing the initial injection molding simulation results to further reduce warpage. In this step, the CAD model of the redesigned, inverse-contoured molded part was imported into Moldflow Insight to conduct a second iteration of the injection molding simulation. The simulation results were exported into ZEISS Inspect software for a final analysis and comparison with the reference CAD model. The warpage values after inverse contouring were reduced within the range of ±0.30 mm, which represents a significant decrease in warpage of approximately 82%. Both steps are presented in a case study on an injection molded part made of polybutylene terephthalate (PBT) with 30% glass fiber (GF).

## 1. Introduction

Injection molding is a versatile and widely used method for manufacturing polymer products. Although researchers have studied the optimization of the injection molding process for decades, molded parts are often manufactured with defects that influence their properties (optical, mechanical, etc.), dimensions, and shape. One of the most common molded part defects is warpage. Essentially, warpage is the unwanted deformation of a plastic component that occurs during the cooling phase of the injection molding cycle and, in some cases, after a molded part is ejected. It is caused by anisotropic material properties due to molecular flow and fiber orientation [1,2,3,4,5,6], polymer material crystallization behavior [7,8,9,10,11], thermal variations within the mold [12,13,14,15], the release of residual stresses in the material [16,17,18,19,20,21], or uneven shrinkage inside a mold cavity because of uneven cooling of the part due to its complexity [12,22,23]. A common approach to mold design is to match the geometry of the cavity to the part shape [24,25,26,27,28,29,30,31] and optimize the mold runner, cooling systems, and the most significant injection molding parameters [32,33,34,35,36]. Some causes of molded part warpage can be predicted using contemporary simulation tools [37,38,39,40,41,42,43,44,45,46]. However, despite these efforts, trial-and-error cases occur in practice.

This study aims to minimize warpage using a two-step approach:

(a) Optimization of processing parameters through an injection molding simulation based on the Response Surface Methodology (RSM) to reduce part warpage;
(b) Inverse-molded part modeling to adjust the part geometry in critical zones, followed by alignment of the warped geometry with the reference model using inspection software to improve dimensional accuracy.

The novelty of this method is the application of software for injection molding simulation and inspection. Inspection software is usually used for a comparison of scanned, manufactured parts with a reference geometry; however, this study presents an approach for achieving a more accurate alignment of the digital warped and reference molded part geometries. Although the warped/opposite part geometry can be applied directly within simulation software, inspection software enables a more accurate alignment and detection of geometry deviations. While simulation software allows for exporting geometries in a solid CAD format, the automatic generation of the warped/opposite molded part geometries can result in critical zones when the part is ejected from the mold (e.g., undercuts in the part ejection direction). This is another issue addressed in this paper and solved via a manual redesign of the already warped CAD geometry.

## 2. Molded Part Warpage

### 2.1. Warpage—Definition

Warpage is a critical defect in injection molding that leads to dimensional and shape deviations in molded parts [12,13,14,23,25,37,38,39,40]. It is often unavoidable, particularly in components with complex geometries or when processing reinforced polymers [6,23,41].

Warpage is a common defect in injection molded parts, caused by non-uniform residual stresses [12]. It typically results from several factors [12,13,30,31]:

(a) Uneven shrinkage between local areas: Temperature variations across different regions of the part cause differences in polymer crystallization (in the case of semi-crystalline polymers) and inconsistent shrinkage. In particular, differences in shrinkage along the thickness of the part can cause distortion and warping.
(b) Non-uniform cooling: Some polymer molecules solidify early with minimal shrinkage, while others remain molten longer. After mold ejection, these areas continue to cool without the restraint of the cavity, resulting in warpage, especially in areas where cooling is slower.
(c) Differential shrinkage from molecular and fiber orientation: In reinforced plastics, the orientation of polymer chains or fibers during flow can create internal imbalances in mechanical properties. This often leads to reduced strength in the direction perpendicular to the flow, contributing to deformation.
(d) Uneven heat dissipation, such as the “corner effect”: The accumulation of material at corners, or the use of different materials or inserts in the mold, can lead to variations in localized cooling and polymer crystallization. These inconsistencies further exacerbate warpage.

Residual stresses primarily arise from two sources. Flow-induced stresses result from the alignment and orientation of polymer molecules as the polymer melt is injected and packed into the mold cavity. Thermal-induced stresses develop when the polymer melt is cooled [17,18,19].

Warpage has been used as an indicator of residual stresses [18] and the anisotropy of mechanical properties [13] in a large number of studies.

Chen et al. [20], Acevedo-Morantes et al. [21], Kim et al. [22], and Tang et al. [42] similarly considered cooling temperature and time in their analyses of residual stresses in plastic injection parts. Also, Guevara-Morales et al. [18] showed that the processing parameters that were most influential on residual stresses and warpage can serve as indicators of the residual stress phenomenon, and vice versa. From a product quality perspective, warpage compromises dimensional and shape stability, causing parts to fail to meet design specifications, which potentially impairs their functionality and assembly and results in them becoming waste products [24,32,47].

Therefore, comprehensive studies focused on a better understanding of warpage, its main causes, and strategies to minimize or eliminate it are briefly presented in Section 2.2.

### 2.2. Warpage—The Main Sources

**Figure 1:**
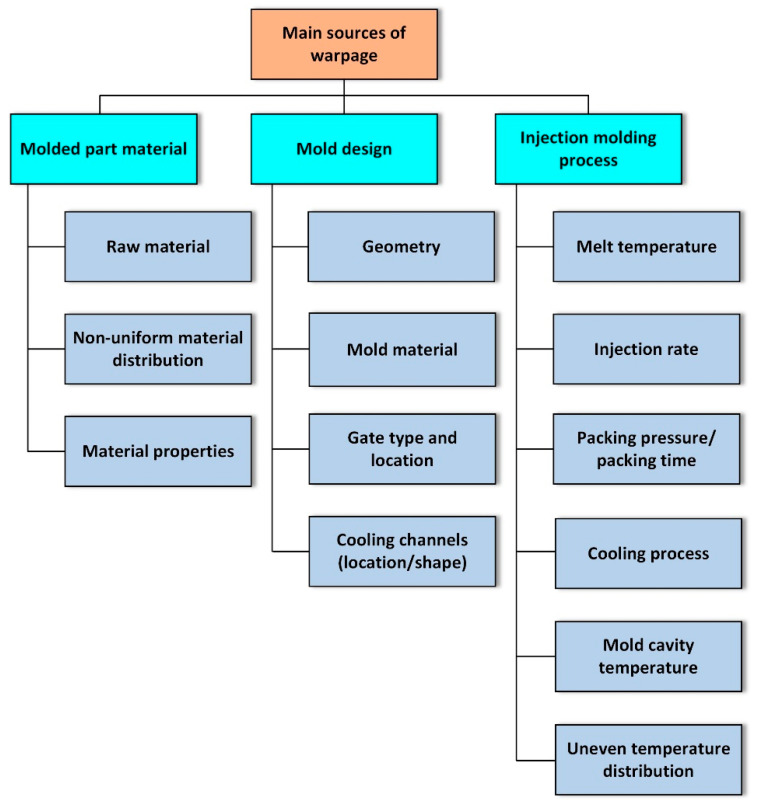
Main causes of molded part warpage (adapted from [13], SAGE Publications, 2024).

Extensive research on injection molding process parameters over the past decades has resulted in the widely accepted conclusion that molded part warpage results from three main causes: the polymer material of the molded part, mold design, and injection processing parameters [13] (Figure 1). This study mainly focuses on analyzing the influence of the mold design and injection molding parameters on molded part warpage.

#### 2.2.1. Influence of Polymer Material on Molded Part Warpage

Semi-crystalline plastics are generally more prone to warping than amorphous plastics. Factors like variations in molecular weight and the entanglement of polymer chains limit the ability of a polymer melt to achieve a fully periodic, crystalline arrangement. As a result, the material exhibits both crystalline and amorphous regions with different physical properties and shrinkage [13,33,34].

Semi-crystalline materials exhibit anisotropic flow because of the complex interaction between molecular orientation, flow-induced crystallization, and morphology development. During the flow of polymer melt, molecular chains are oriented in the flow direction, which reduces viscosity in the flow direction, resulting in anisotropic rheological behavior. Flow-induced molecular chain orientation accelerates crystallization kinetics, especially in the flow direction, increasing the anisotropy of the polymer morphology and mechanical properties. The formation of a so-called skin–core structure is an additional mechanism resulting in increased polymer flow-induced anisotropy. During injection molding, the skin layer, which is in contact with the mold surface, is under high shear, which induces a highly oriented fine crystalline structure and increases the crystallization rate compared to the core region. This leads to the local anisotropy of thermal and mechanical properties, which can further cause anisotropic shrinkage and thus enhance the warpage of injection molded parts [7].

Crystallization also occurs during cooling and is time- and temperature-dependent. Slower cooling (due to high cavity surface temperatures) leads to a higher degree of crystallization and shrinkage, increasing the potential for high stresses and warpage. If the temperature decreases too rapidly, nucleation and nucleus growth can be suppressed. This will increase the proportion of the structure with a low degree of crystallization, resulting in lower molding shrinkage. This can then lead to more pronounced post-crystallization and thus undesirable post-shrinkage [4,34,44,48,49].

Adding fibers to a plastic material can influence shrinkage behavior by counteracting the effects of molecular orientation [50]. Fiber-reinforced materials typically exhibit better mechanical properties in the longitudinal direction than in the transverse direction—primarily due to the alignment of the fibers [43,49].

Zöllner et al. [34] concluded that, with the use of glass fibers, it is possible to reduce shrinkage by 50 to 80% in the longitudinal fiber direction.

#### 2.2.2. Influence of Injection Molding Parameters on Molded Part Warpage

**Figure 2:**
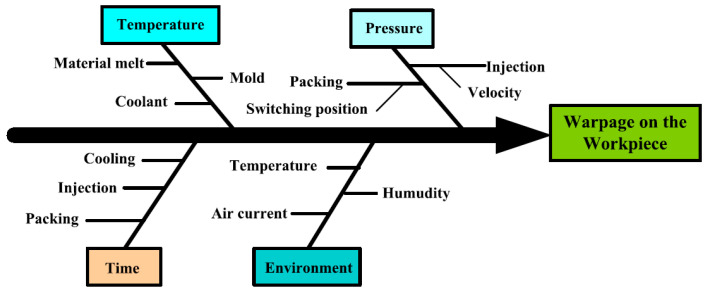
Fishbone diagram of warpage (adapted from [37], Springer, 2025).

Incorrect estimation and improper setting of key injection molding parameters can lead to an increased warpage of molded parts. In general, the most significant injection molding parameters affecting warpage are categorized in Figure 2 [37]. With the exception of environmental factors, the remaining parameters and their impact on molded part warpage will be described in more detail in the following sections.

*Mold temperature (cavity wall)*

Mold temperature affects injection molding productivity and the quality of molded parts. Maintaining an appropriate mold cavity temperature field can improve the mechanical properties, surface quality, and dimensional and shape accuracy and reduce the warpage of molded parts. Moreover, this can improve productivity by shortening the cooling time of molded parts [12].

For warpage, it is crucial that the mold cavity temperature is reached and maintained as uniformly as possible in all mold cavity zones [12,26,35,46,51,52,53,54].

Mold temperature can affect how much residual stress is generated and the degree of crystallization. An elevated mold temperature can result in reduced molded part warpage, but it will lead to longer cooling times [12,33,55]. Increasing the mold temperature without prolonging the cooling time results in worse molded part properties [33,56,57].

Temperature variations within the mold can significantly contribute to the warpage of injection molded parts, primarily due to the induction of residual stress gradients. These stress imbalances often induce bending, which manifests as molded part warpage upon ejection from the mold [15,33,55,57].

*Melt temperature*

An insufficient melt temperature leads to an increased melt viscosity, requiring higher injection pressures and potentially resulting in short shots due to premature gate freeze before the cavity is completely filled. Moreover, low melt temperatures can contribute to elevated residual stresses within the molded part, which may promote warpage during its cooling and solidification [5].

Increasing the melt temperature up to a certain point decreases shrinkage due to decreased viscosity and a better distributed packing pressure throughout the cavity [34]. For a given packing pressure and time, further increases in the melt temperature lead to higher shrinkage, as the cavity is filled with relatively low-density material [58,59].

However, if the melt temperature is too high, the material can easily overheat and degrade, causing it to shrink greatly after cooling to room temperature [4,34,44].

In summary, the melt temperature must be carefully optimized within an appropriate range to ensure optimal processing conditions and the desired quality of the molded part [5,12,34,58,59].

*Cooling time*

To avoid deformation, molded parts need to cool down and become rigid enough before they are removed from the mold cavity. How much stress or warping occurs during ejection depends on the molded part geometry, ejection system, shrinkage level, and friction between the molded part and the mold cavity surfaces [59].

A longer cooling time allows the part to become stiffer, which can reduce warpage. Since molded parts retain their shape in the mold cavity for an extended time, prolonging the cooling time can minimize molded part warpage [4,59]. For a rough estimation of the cooling time of molded parts, the temperature derived from differential scanning calorimetry (DSC) measurements on the cooling melt can be used as the demolding temperature when processing semi-crystalline materials [60].

*Coolant temperature*

The mold coolant temperature is the temperature of the fluid (typically water or oil) used to control the mold cavity wall temperature during the injection molding process. This parameter plays a key role in achieving the desired molded part quality, injection molding cycle time, and molded part properties [61]. As a general guideline, the coolant inlet temperature should be about 10 to 20 °C lower than the target mold temperature. To achieve uniform mold cooling, it is also recommended that the temperature difference between the inlet and outlet stays within a range of 2 to 3 °C [27,62].

*Injection speed*

If the injection speed is too low, regions of the polymer part may begin to solidify before the mold cavity is filled, causing uneven shrinkage, deformation, and short shots. A lower polymer melt injection speed can also result in a lower initial melt pressure and potentially less effective packing, resulting in voids and an uneven molded part density, and, as a result, the molded part is more prone to warp. An insufficient injection speed can make processing more difficult, especially when the mold temperature is low, which reduces the polymer melt flowability [63].

However, higher injection speeds generate additional shear heat, which counteracts the cooling effect and facilitates polymer melt flow through the mold cavity. In general, increasing the injection speed slows down heat loss from the polymer, improving mold cavity filling [63]. The mentioned considerations should be taken into account, particularly during the simulation of injection molding.

The injection speed also influences the degree of molecular orientation in the polymer melt [34,59]. At higher speeds, increased shear forces cause polymer chains or fibers to align more in the direction of flow in the skin region, and, in the core, the fountain flow effect can lead to chain alignment that is perpendicular to the flow [63].

*Injection pressure*

A higher injection pressure can increase the differences in the molecular and fiber orientation within plastic parts, leading to greater residual stresses and a higher risk of warpage. In contrast, using a lower injection pressure tends to reduce the molecular orientation and minimize residual stresses. To reduce warpage, the injection pressure should be set as low as possible while still ensuring proper mold filling [12,64].

*Packing pressure and packing time*

The interaction between the packing pressure and packing pressure time, and their optimal combination, is essential for controlling molded part warpage.

During injection, the cavity pressure is the highest closest to the gate. This means that the melt in this area shrinks less than the plastic melt located farther away. An optimized packing pressure ensures that sufficient plastic melt flows into the mold to fill it and tightly compresses the product, contributing to lower shrinkage during cooling cycles. With lower overall shrinkage, the warpage of the plastic part can be reduced by increasing the cavity pressure [4,27].

If the packing pressure is too high, it can generate significant flow-induced residual stresses in the part, leading to warpage after ejection. However, if the packing pressure is too low, backflow can occur near the gate, causing residual shear stresses and molded part warpage [4].

The packing time must also be carefully optimized to ensure both high product quality and efficient production. If the packing time is too short, the molded part may not be fully compressed. Once the gate has solidified, extending the packing time no longer improves the molding quality and unnecessarily prolongs the cycle time [4,27].

#### 2.2.3. Injection Molding Parameter Optimization for Minimizing Warpage

The above-mentioned injection molding parameters and their optimization for minimizing warpage have been studied by many researchers. Researchers have applied different research methods (simulation and experimental), observed different injection molding parameters, and obtained different results regarding the most significant parameters affecting molded part warpage. Table 1 summarizes the results of relevant research.

Most of the referenced studies successfully achieved significant warpage reduction in injection molded components by either enhancing the mold design or optimizing the processing parameters through various optimization techniques. As the mold design and processing conditions are both critical factors affecting molded part warpage, focusing on only one aspect may yield suboptimal results. Consequently, a combined approach that simultaneously addresses both the mold structure and the process parameters is likely to result in more substantial reductions in molded part warpage.

Furthermore, researchers have reported varying results and drawn differing conclusions regarding the most influential injection molding parameters affecting warpage (Table 1). Therefore, there is no universally applicable conclusion regarding the most critical injection molding parameters influencing part warpage. Instead, each study should focus on specific injection molding parameters and their interactions based on the research goals of that particular study.

#### 2.2.4. Influence of the Mold on Molded Part Warpage

Considering mold factors, such as geometries and materials, is essential for optimizing warpage. The selection of materials with higher thermal conductivity for mold core and cavity zones exposed to higher heat concentrations reduces the temperature differences in other zones of the mold cavity wall. Faster cooling in these critical zones also reduces warpage caused by different cooling rates [9,32,81,82,83]. Lucyshyn et al. [84] found a positive effect on warpage reduction when higher-thermal-conductivity mold materials were applied. Azli et al. [85] analyzed the simulation results of the molding cycle time and molded part warpage using tool steel, aluminum alloys, and copper alloy inserts with conventional cooling channels. Aluminum alloys were selected as the best combination for minimizing the cycle time and warpage.

The gate type, dimension, and location have direct impacts on cavity filling and the properties of molded parts, especially in the processing of reinforced polymers. Areas of the molded part that are closer to the gates typically experience less shrinkage due to more effective packing. If the gates are too small, they may solidify too early, preventing the packing pressure from reaching the far end of the cavity, resulting in an increased risk of molded part warpage [27,32,44].

Additionally, if the gates are too small, the material experiences high shear stress as it flows through the gate, which can further contribute to part warpage [4,44].

The longer it takes for the melt to flow through the mold cavity, the higher the probability of developing orientation stress. To minimize this, for molded parts with thick walls, long flow paths, and/or large surface areas, it is beneficial to use multiple gates to reduce warpage. However, using multiple gates can also increase the chances of visible weld lines [4,44].

There are many methods for optimizing the number of gates and their arrangement [42,86,87,88,89,90]. These methods are aimed at ensuring the best possible molded part properties and improving the injection molding parameters. With an appropriate gate arrangement, it is possible to shorten the time it takes to fill the mold, improve the mold’s cooling efficiency, and reduce warping.

The design of the cooling system also plays a critical role in controlling molded part warpage. The mold cooling system must efficiently remove heat while maintaining a uniform temperature distribution across the mold and molded part. The performance of the cooling system can be evaluated by analyzing the heat flux distribution, which indicates the rate of heat removal per unit area and reveals variations in cooling efficiency across different regions of the mold. High heat flux is desirable for reducing the cycle time, but a poor cooling system design can lead to an uneven temperature distribution in the mold cavity, causing molded part warpage after ejection [27].

The positioning, size, and shape of the cooling channel are essential mold variables to guarantee uniform cooling, reducing the temperature gradients that cause residual stresses. Properly designed cooling channels improve the cooling performance, reduce the cycle time, and ensure a uniform thermal distribution across the molded part [32,91,92].

In many cases, improving the layout and design of cooling channels helps reduce molded part warpage. However, this approach is often limited by the complexity of the mold design and the restricted space for the placement of cooling channels [32].

When designing a cooling circuit, the goal is not only to maintain a uniform mold temperature field but also to ensure a uniform cooling time across the molded part. Higher mold temperatures promote more uniform shrinkage and reduce the molecular orientation, but they also require extended cooling times [4].

Isler et al. [32] examined how the diameter of cooling channels affects the warpage of molded parts. Simulation results revealed that cooling channels with a 6 mm diameter led to increased warpage, whereas 8 mm and 10 mm channels resulted in lower warpage. The 8 mm cooling channel diameter resulted in the least warpage.

Today, advancements in manufacturing technologies have expanded the possibilities for cooling channel design. For example, 3D printing enables the creation of conformal cooling channels (CCCs) by allowing mold inserts to be built layer by layer [93]. Unlike traditional straight-line channels, which often result in uneven distances from the mold surface and irregular temperature distributions, CCCs follow the exact contours of the mold. This ensures a consistent distance between the cooling channels and the mold surface along their entire length. As a result, CCCs provide a more uniform temperature distribution and cooling, helping reduce residual stresses and differential shrinkage [38].

The researchers in [67,83,94,95,96,97] showed that using CCCs instead of traditional straight channels reduced molded part warpage by 32% to 48%.

### 2.3. Minimizing Molded Part Warpage by Inverse Mold Design

As already mentioned, frequent changes in the processing parameters alone are insufficient for warpage mitigation. However, the application of conformal cooling requires the application of additive manufacturing, which is not always available. Moreover, cooling channels with diameters larger than 8 mm require the application of support structures within the channels, and it is very difficult or even impossible to remove those structures. Therefore, a few studies have analyzed the possibilities of shaping molded parts intentionally in the opposite direction of estimated warpage.

Zhai et al. [98] applied several optimization methods and developed an algorithm for an inverse design of the mold cavity for a box-shaped part. A method is used to compare the positions of each mesh node between the reference and deformed geometries. In each iteration, the cavity geometry is adjusted in the opposite direction of the deviations between the deformed and reference geometries. The authors used a multi-objective optimization algorithm for six molding parameters. Following process optimization, software was used to simulate the injection process, and the exported displacements were used to design the mold cavity. This method was validated via optical scanning of the molded part and proved to be very effective.

**Figure 3:**
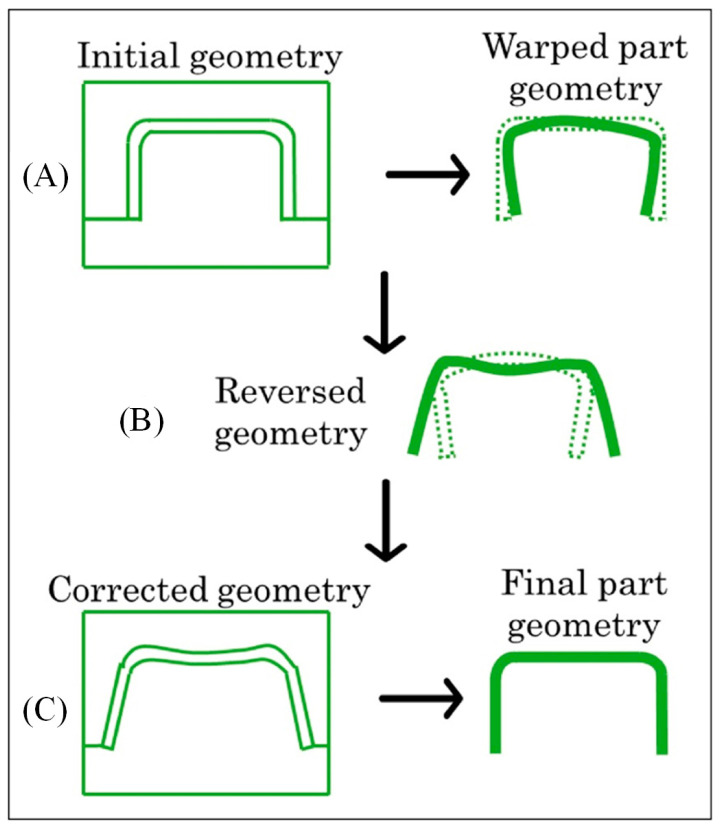
Schema for correcting geometry to reduce warpage: (**A**) initial mold geometry results with warped molded part; (**B**) reverse molded part modeling in opposite direction, (**C**) designing mold based on reversed geometry results with final molded part geometry with minimal warpage (adapted from [99], Wiley, 2024).

Tillmann et al. [99] introduced Bayesian optimization, which includes multiple iterations, as it uses a global objective function. The authors implemented regression to reduce and optimize the warpage analysis via the finite element method (FEM), modifying the original shape of the mold to avoid molded part warpage (Figure 3). The above-mentioned methods require integration into the software’s source code, which is, to some extent, a drawback.

Another numerical method presented by Katelic et al. [100] goes further than using the warpage displacement field by making iterations of simulations on deformed parts. This method uses local deviations to correct the mesh nodes and calculate the new mold cavity shape in each step, thereby making the entire process more robust. The implementation of the method in a simulation in two case studies showed a reduction in warpage of more than 90%.

Zwicke et al. in [29,101] introduced an inverse design method focusing on thermo-elasticity applying an approach different from shape optimization. The method, initially developed by Zwicke in [102], involves performing an inverse warpage simulation. The desired mold cavity shape is used as input, and the unknown mold cavity shape is the output. He divided the simulation process into two steps: a solidification simulation (for molded part ejection shape determination) and a shrinkage and warpage simulation (for warped molded part shape determination, consisting of temperature and residual stress distributions). The method consists of a forward simulation to calculate the temperature distribution before molded part ejection. For the inverse step, this temperature distribution is used as the initial data to calculate the modified mold cavity shape. This method is simple and inexpensive, requiring only one forward and one inverse run of the model.

Tilmann et al. in [103] applied the reverse geometry method for warpage compensation in changing meshes with interpolation methods. The authors showed that it is possible to apply the presented method in simulations of models that remesh the geometry. Another reported advantage over the method presented in [100] is simplicity and fewer iterations required for optimal results.

Tilmann et al. in [104] compared numerical methods for warpage compensation through an analysis of five different part designs. The chosen parts were good examples of the geometries and topologies of plastic products. The results showed that the inverse design method [100,101,102,103] is most efficient in terms of the number of iterations and significance of molded part warpage control.

The method used in this paper is comparable to that used in both studies performed by Katelic et al. [100] and Zwicke et al. [29,101]. For the purpose of this study, the abilities of Autodesk Moldflow Insight software to predict warped molded part geometries and, in particular, to automatically generate the opposite (inverse) geometries of warped parts were utilized without performing the additional computation and iterations presented in [29,100,101,102,103]. This paper explains the process of inverse contouring, a technique used to design mold cavities so that the areas of a molded part expected to warp significantly are intentionally shaped in the opposite direction. This ensures that, after warping occurs on such an inverse deformed geometry, these areas shift to their intended final position, satisfying the tolerance requirements and ensuring that the part functions as intended. The effectiveness of this method can be validated more accurately by aligning the warped inverse-contoured geometry with the ideal, reference CAD geometry.

## 3. Materials, Methods, and Results

**Figure 4:**
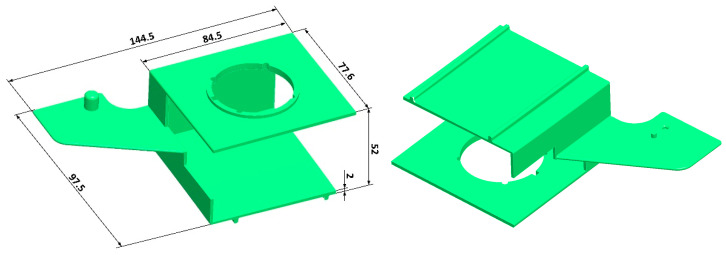
Reference molded part CAD geometry.

The part analyzed in this study (Figure 4) is made of 30% glass fiber-reinforced polybutylene terephthalate PBT-GF30 (trade name: Pocan B3235; ISO short name: ISO 20028-PBT, GF30, GHMR, 09-100; manufacturer: Envalior GmbH, Düsseldorf, Germany). This material, intended to be used in injection molding, is commonly used in the automotive, electrical, and electronics industries due to its flowability, heat resistance, and ability to be treated with flame retardants. Table 2 gives an overview of the relevant properties of the applied material.

A CAD model of the molded part (Figure 4) is designed with CAD software PTC Creo 2.0 (PTC Inc., Boston, MA, USA). The main dimensions of the molded part are shown in Figure S1 (Supplement). The characteristic molded part wall thickness is 2.0 mm.

The inverse contouring method is demonstrated on a specific case of an already manufactured, non−optimized mold, which implies certain limitations in the gate and cooling channel positions. For manufacturing, a single cavity mold is designed. The main characteristic of the mold design is the application of a mechanically actuated (by an angled pin) slider with a stroke of 72.5 mm and a cold runner system with a tunnel gate.

The inverse contouring method is conducted in two main steps. In the first step, a computer simulation and RSM are used for the initial optimization of the cooling channels and processing parameters, followed by the second step in which the molded part is inverse−contoured for additional warpage minimization.

In this case, the inverse contouring method process can be described as follows:

Step 1. Simulation and RSM warpage optimization

1.1 Initial injection molding simulation with the reference molded part;
1.2 Statistical warpage analysis and optimization (RSM);
1.3 Alignment and validation of the warped and reference molded parts.

Step 2: Inverse contouring warpage optimization

2.1 Molded part inverse contouring (redesign);
2.2 Inverse−contoured molded part injection molding simulation;
2.3 Warped inverse−contoured and reference molded part final alignment and validation.

### 3.1. Simulation and RSM Warpage Optimization

#### 3.1.1. Initial Injection Molding Simulation with Reference Molded Part

The injection simulation activities were performed with Autodesk Moldflow Insight 2023 software (Autodesk Inc., San Francisco, CA, USA). In the first step of the simulation process, a finite element mesh was generated. A high-quality, dense mesh is critical for the desired results of inverse contouring to be achieved. In all the simulations presented in this paper, the part geometry was meshed using approximately 830,000 tetrahedral elements.

**Figure 5:**
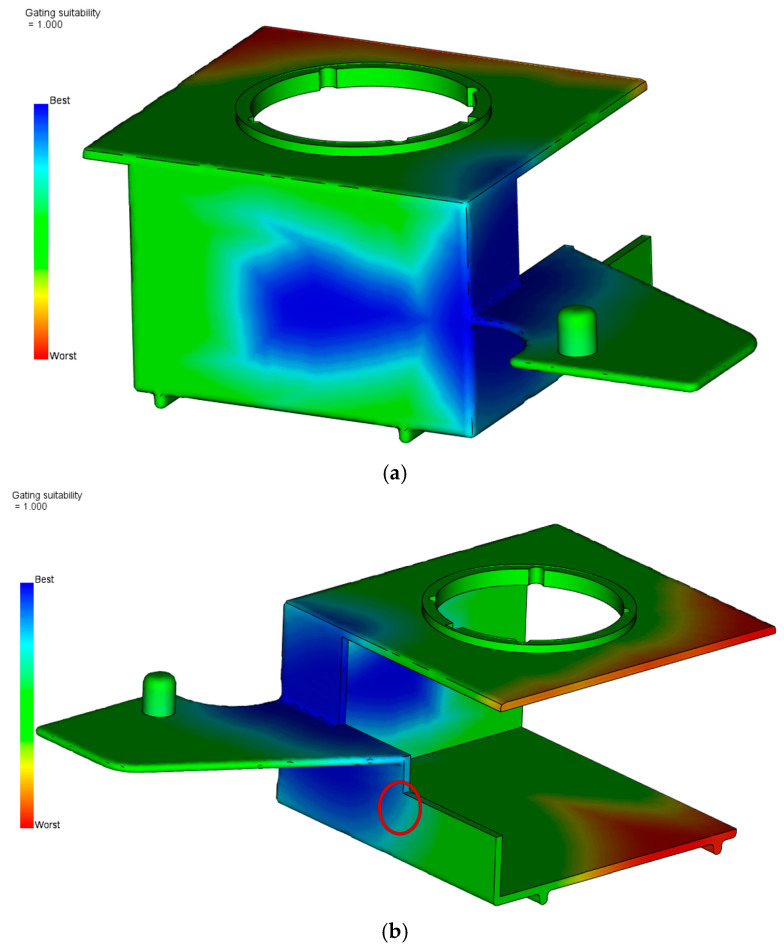
Analysis of the best gate locations (blue areas are acceptable): (**a**) external back location, (**b**) external side location (red circle marks the location of actual gate).

Although a mold was already designed and manufactured in this case, a preliminary analysis of the best gate position was performed. The main criterion for the calculation was the melt flow resistance obtained by the wall-thickness-to-flow-length ratio. Figure 5 shows the results of this analysis.

**Figure 6:**
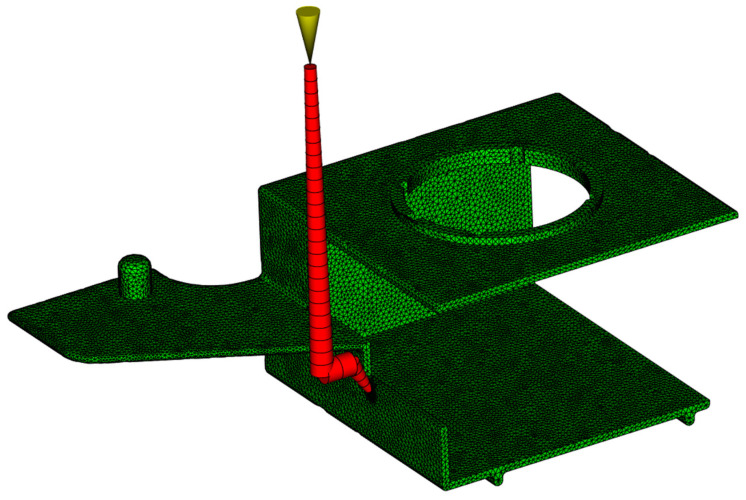
Meshed part geometry in Autodesk Moldflow Insight with visible runner system.

Although there are two main suitable areas suggested, the gate location in the area presented in Figure 5a requires larger mold dimensions and, consequently, the manufacture of a completely new mold. In the case of the side area, as shown in Figure 5b, because of the parting plane orientation (presented in Figure S1—Supplement) and the ejection of the runner systems, the actual gate should be located more to the left from the best option (marked with a red circle), which is also in correlation with the manufactured mold (the movable mold plate design presented in Figure S2—Supplement). Figure 6 shows the meshed molded part geometry with the injection point location defined for all performed analyses. The area between the mold cavity surfaces is filled with approximately 8 tetrahedral elements through the thickness of the part with a total of 632 elements.

The simulation model included a mold base, as well as cooling channels. The part was set into a steel mold (1.2343) with dimensions of 246 × 296 × 176 mm. The cooling channels were 8 mm in diameter and located both above and below the molded part, as well as within the slide shaping internal molded part area, with a coolant temperature of 60 °C (20 °C below the expected mold temperature, as recommended in [62]).

**Figure 7:**
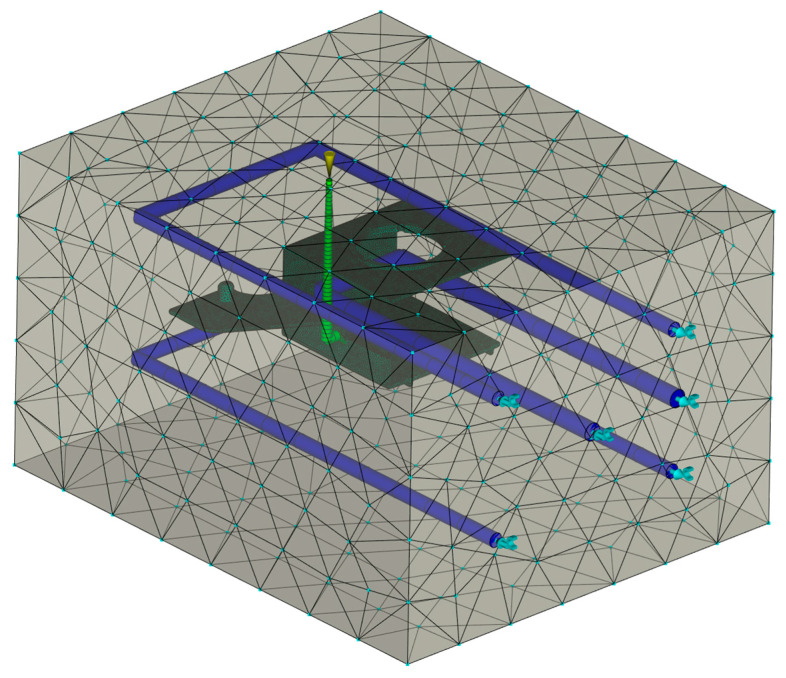
Simulation model with mold surfaces and cooling channels.

The coolant used was water with a Reynolds number of 10,000, ensuring turbulent flow for optimal heat exchange within the mold. Several studies with different mesh sizes were conducted to ensure that the results were mesh-independent. Table 3 contains the main boundary condition data of the simulation model, and Figure 7 shows the simulation model used in the following analyses.

For the purpose of this study, a Cool + Fill + Pack + Warp sequence of simulations was performed in Autodesk Moldflow Insight. The Cool analysis calculated the heat exchange within the mold using the boundary element method [107]. Performing this analysis is recommended for geometry optimization tasks.

Fill + Pack analyses solve the melt flow inside the mold cavity via Navier–Stokes equations for non-Newtonian viscosity [107]. All the flow equations were solved using the standard finite element method. Parameters, such as temperature and pressure, were solved iteratively until convergence was achieved. These analyses gave insights into the cycle time, injection pressures, clamp force, melt temperature at certain points and at a given time, fiber orientation, etc.

The final analysis in the sequence was the Warp analysis, which solved part warpage upon ejection of the molded part from the mold cavity. When working with fiber-reinforced materials, it is essential to enable the fiber orientation analysis in the Fill + Pack analyses, as it has a significant impact on the warpage results. The Warp analysis uses the pressure and temperature fields from the previous stage to calculate changes in the part geometry, which are caused by residual stresses that develop due to temperature and pressure variations during ejection from the mold cavity.

**Figure 8:**
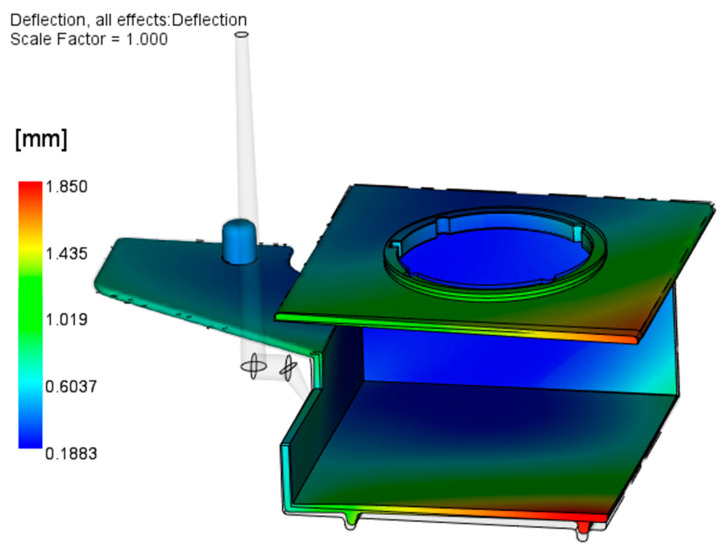
Results of the initial Warp analysis.

Figure 8 shows the results of the initial Warp analysis, conducted with the reference CAD geometry without any modifications. The results indicate a tendency of the large horizontal surfaces of the parts to warp toward each other.

**Figure 9:**
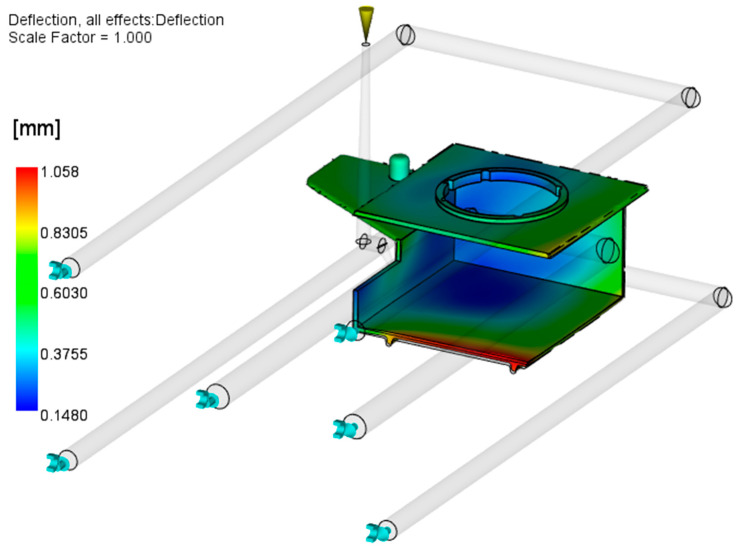
Influence of increasing the slide cooling channel diameter from 8 to 10 mm on warpage.

**Figure 10:**
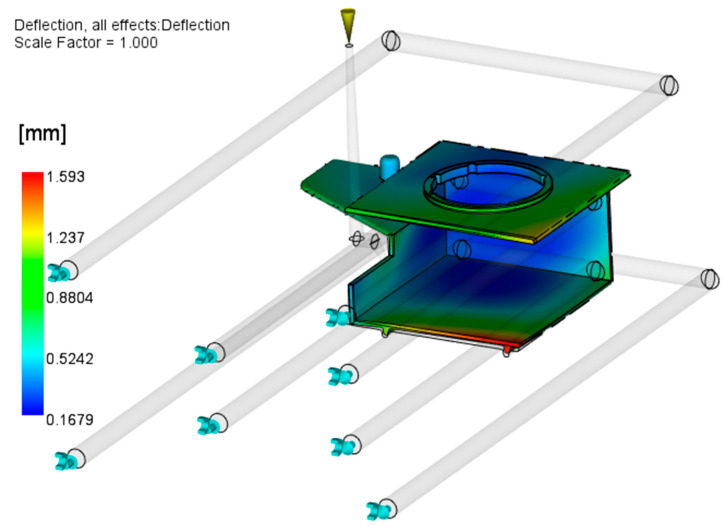
Influence of applying two cooling channels (8 mm) on warpage.

From the results presented in Figure 8, it can be concluded that the molded part geometry (open box-like shape) and inadequate injection molding process parameters and mold design are the main causes of excessive warpage. The optimization of the injection molding parameters will be described in detail in Section 3.2; here, the focus is on the attempt to optimize the mold cooling setup. As the part is warped inward from both (upper and lower) sides, it can be concluded that the slider is hotter than the cavity plates. The optimization of the slide cooling channels is performed in two steps: (a) increasing the diameter of the existing channel from 8 mm to 10 mm (Figure 9) and (b) applying two 8 mm channels moved by 15 mm from the middle slide section in both directions (Figure 10). The processing parameters used are given in Table 3.

Figure 9 presents the influence of the increased internal slide cooling channel on the molded part warpage. It is obvious that increasing the cooling channel diameter improves the heat flux from the cavity and thus decreases the temperature differences from the slide and mold cavity plates, which causes warpage to decrease (1.06 mm).

Figure 10 presents the results of applying two 8 mm cooling channels. Although the cooling channels are at smaller distances to the cavity wall than in the application of only one channel, the simulation results show that this design results in a larger maximal warpage value of the molded part (1.59 mm).

Therefore, one cooling channel with a 10 mm diameter was used for further warpage optimization steps.

The tunnel gate dimensions were already set in the initial mold design, and the smallest gate diameter was set to 1.5 mm according to the recommendations in [106]. Therefore, the gate dimensions were not changed in further analyses.

#### 3.1.2. Statistical Warpage Analysis and Optimization (RSM)

**Figure 11:**
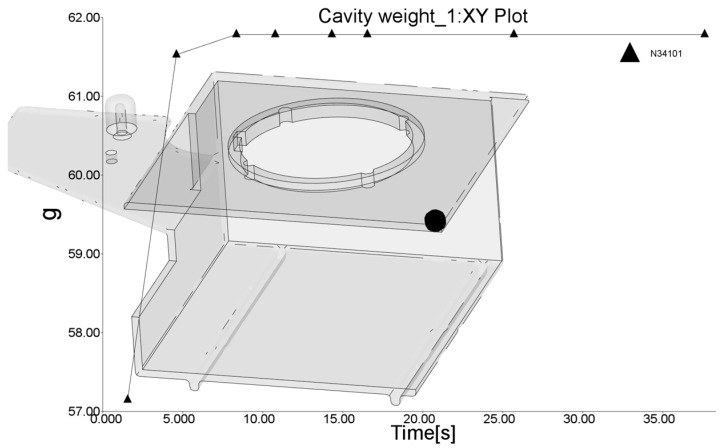
Estimation of packing time (10 s).

The RSM optimization of the injection molding parameters for minimizing warpage was performed by analyzing three parameters: the melt temperature, target mold temperature, and coolant inlet temperature in the slide cooling channel. The packing pressure and packing time were also considered significant processing parameters, but, in this case, they were maintained at maximum levels, as a number of previous studies [31,65,70,73,78,79,80] showed that elevated packing pressure levels and longer packing times have positive effects on decreasing warpage. A value of 150 MPa was set for the packing pressure, and, for the packing time, 10 s was set according to an analysis of the time needed to reach the final molded part weight at the longest distance from the gate (the black dot in Figure 11).

For a statistical analysis of the influence of the above-mentioned processing parameters, RSM, namely, the central composite design (CCD), was applied. The CCD in general consists of 2^k^ runs on the peak values of the observed parameters, 2k runs in the axes of each parameter, and trials at the center of the design (*k* is the number of adjustable parameters) extended with additional runs in the center of the design and the axis of each parameter to be able to estimate parameters with second-order models.

The desired characteristic of each design of experiment (DoE) is an independent estimation of the main factors and their interactions, which is a function of the rotatability of the design [108]. A design is rotatable if the following relationship exists [109]:

α = ∜F(1)

where α is the distance of a run in the design axis from the center of the design, and F is the number of parameters.

In the case of a CCD with three adjustable parameters, F is equal to 8; therefore, the α value is 1.68. Therefore, the design factors in the coded form can be presented as −1.68, −1, 0, 1, and 1.68. In that case, the CCD consists of a total of 15 runs: 14 at the design peaks and along the axes and 1 at the design center.

The melt temperature, target mold temperature, and slide channel inlet coolant temperature were varied according to the CCD for optimization, as shown in Table 4. The values are arranged based on the polymer producer data and recommendations [105]. In [106], it is also recommended that the inlet coolant temperature be set in a range 10 to 20 °C below the target mold temperature. Therefore, the coolant temperature was set to 70 °C as a middle value, and other variations were made according to the rules of the selected CCD.

Table 4 also shows the maximal values of molded part warpage as a result of the Autodesk Moldflow Insight simulation.

A graphical presentation of the simulation results of the warpage analysis is shown in the Supplement (Table S1).

For a statistical analysis of the results, the software Design Expert v. 23.1.7. (Stat-Ease Inc., Minneapolis, MN, USA) was used. An ANOVA and RSM were used to determine the significance of the adjustable injection molding parameters and their interactions on molded part warpage (Table 5).

**Figure 12:**
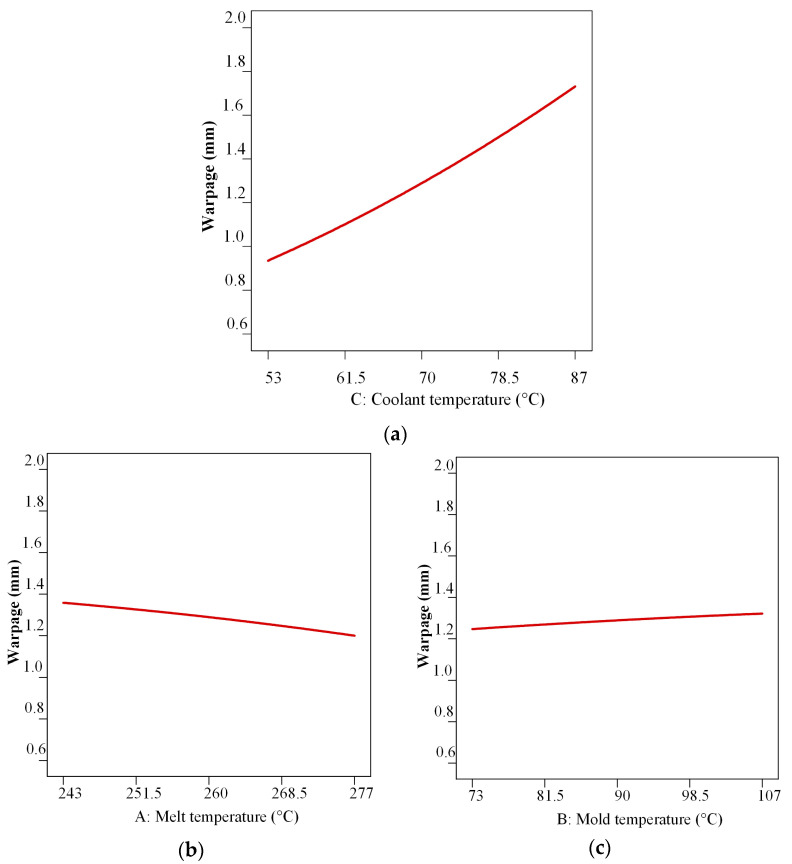
Average effects on molded part warpage: (**a**) coolant temperature, (**b**) melt temperature, (**c**) mold temperature.

In Table 5, it can be concluded that the coolant temperature makes the most significant contribution to the changes in the molded part warpage, followed by the melt temperature and mold temperature. The effects of these parameters on the molded part warpage are presented in 2D graphs in Figure 12.

From Figure 12, it can be concluded that decreasing the coolant temperature, increasing the melt temperature, and moderately decreasing the mold temperature contribute to the warpage decrease.

To set the analyzed injection molding parameters that will result in minimal molded part warpage, Design Expert software was used for the determination of RSM optimization criteria and injection parameter optimization. The RSM optimization limits, shown in Table 6, were set based on the results from the previous RSM analysis, with the temperature ranges of both limit values expanded by 10 °C compared to the values in Table 4.

The RSM optimization suggested the application of a number of combinations of parameters for minimizing warpage. A melt temperature of 280 °C, mold temperature of 100 °C, and coolant temperature of 40 °C were selected for further analysis, with an expected molded part warpage of 0.57 mm.

**Figure 13:**
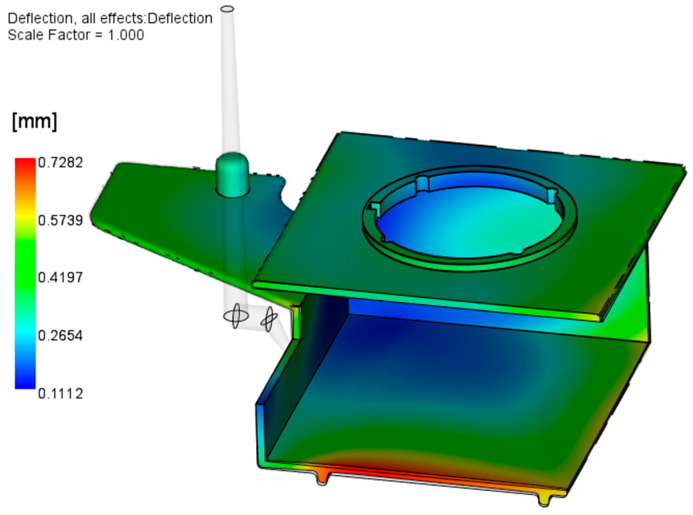
Warpage simulation results with RSM-optimized injection molding parameters.

Therefore, the next injection molding simulation was performed with a set of RSM-optimized process parameters to compare the RSM and simulation results. Figure 13 presents the warpage simulation results.

The maximal warpage simulation result is 0.73 mm, which is higher than that predicted with the RSM optimization. Nevertheless, this value is the smallest warpage obtained in all conducted simulations, and it is approximately 61% lower than the initial simulation result. The warped geometry obtained from this simulation (actual and opposite) will be used for further alignment and inverse contouring.

#### 3.1.3. Alignment and Validation of the Warped and Reference Molded Part

To proceed with the method of inverse contouring, the warped part geometry (the result of the previous simulation with the RSM-optimized injection molding parameters) should be exported from Autodesk Moldflow Insight for a better analysis of the geometry deviations from the reference molded part. This software offers the option to export the part as is (“actual”) or warped in the opposite direction (“opposite”). The actual geometry was exported and loaded into ZEISS Inspect 2023 software (ZEISS AG, Oberkochen, Germany) to be aligned with the reference CAD geometry.

The elements of an Autodesk Moldflow Insight analysis must be sufficiently small to provide enough data for ZEISS Inspect to perform calculations. This is why a high-quality, dense dual-domain mesh in Autodesk Moldflow Insight is important for the inverse contouring method, as the number of triangles in the output STL (standard tessellation language) file, which is uploaded into ZEISS Inspect, is equal to the number of triangles in the dual-domain mesh.

ZEISS Inspect software differentiates between two geometries within a project: the nominal geometry, which is the CAD file with the reference part geometry, and the actual geometry, which, in this case, is an STL file exported from Autodesk Moldflow Insight.

Since Autodesk Moldflow Insight calculates inside the mold cavity, the normals of the elements are directed toward the mold cavity, while, traditionally, the normals of the surfaces of the CAD geometry are directed outward. To ensure that the best alignment is made, it is important to invert the direction of the normal of the imported actual geometry using the Invert Selected Normal function.

Once this correction is made, the Prealignment feature is used to align the actual geometry with the reference CAD data. This feature uses optimization methods, such as least-squares fitting, to reduce the geometric difference between the measured data and the reference model. For the part analyzed in this case, Prealignment provides sufficient accuracy for the inverse contouring method.

**Figure 14:**
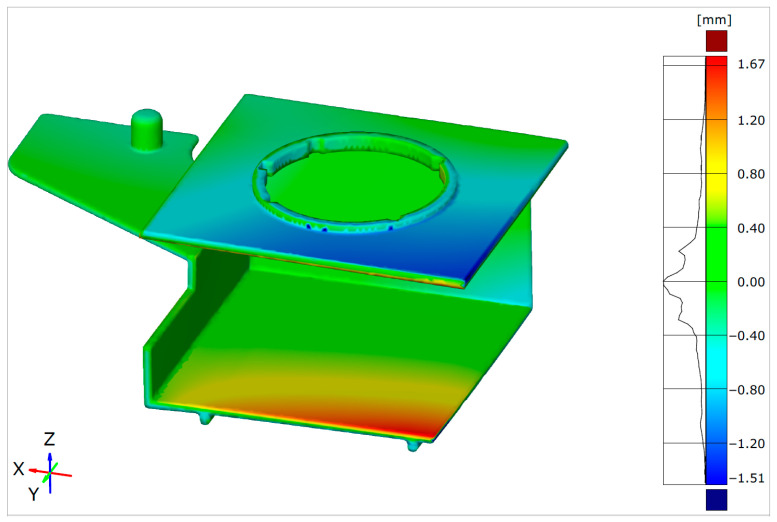
Surface Comparison of initial warped part geometry with reference CAD geometry.

Once the parts are aligned, the Surface Comparison function can give a clearer picture of the deviations between the actual part and the desired geometry. Figure 14 shows the output of the Surface Comparison on the actual geometry for the initially warped part geometry. In Figure 14, the green areas indicate regions where the nominal (CAD) geometry and the actual (warped) geometry are closely aligned. The blue areas show where the actual surface lies below the nominal surface, while the red areas indicate where the actual surface is above the nominal surface. The label on the right side presents the distribution of dimensional deviations.

Comparing Figure 8 and Figure 14, it is obvious that each software indicates two problematic regions, where the ZEISS Inspect software analysis results in a smaller maximal warpage value (1.67 mm) than Autodesk Moldflow Insight (1.85 mm—Figure 8).

**Figure 15:**
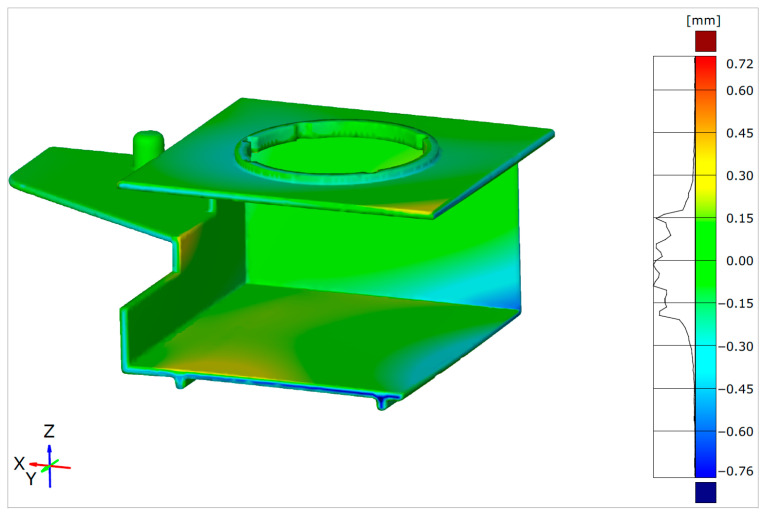
Surface Comparison of RSM-optimized warped part geometry with reference CAD geometry.

The same alignment procedure was performed for the RSM-optimized warped design. The results in Figure 15 show a significant warpage decrease as an effect of injection molding parameter RSM optimization. The maximal warpage value decreased from 1.67 mm to 0.76 mm, which is a reduction of approximately 55% (based on ZEISS Inspect).

### 3.2. Inverse Contouring Warpage Optimization

#### 3.2.1. Molded Part Inverse Contouring (Redesign)

**Figure 16:**
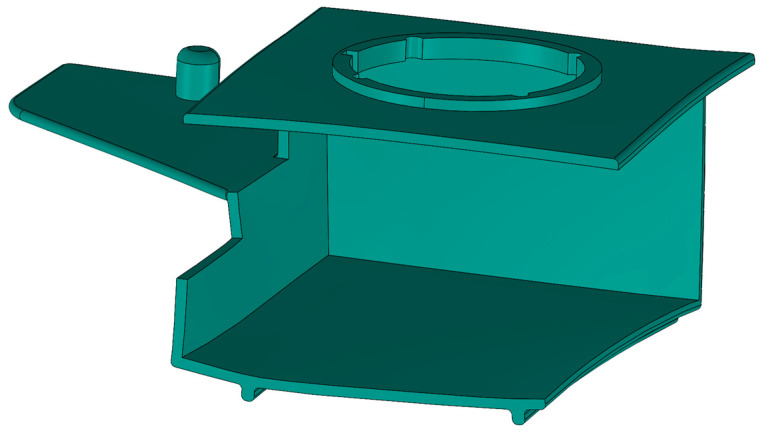
Inverse-contoured molded part CAD model (warpage scale factor 5).

After the initial alignment was finished, the inverse contouring process began by exporting the opposite geometry from Autodesk Moldflow Insight after simulation with the RSM-optimized parameters. As the warped geometry obtained by Autodesk Moldflow Insight included the effects of shrinkage, the exported part was effectively enlarged. Previous versions of Autodesk Moldflow Insight software allowed for exporting warped geometries only as an STL file. In such cases, redesigning the mold for an inverse-contoured molded part required part geometry conversion into a solid body using, e.g., the reverse engineering (RE) method. In this study, Autodesk Moldflow Insight 2023 version was used, which enabled exporting the warped molded part geometry as a full solid model, which, in turn, enabled redesigning the mold core and cavity using standard CAD tools without the need for RE. Figure 16 shows an inverse-contoured molded part with warpage increased by scale factor 5 for better visualization.

**Figure 17:**
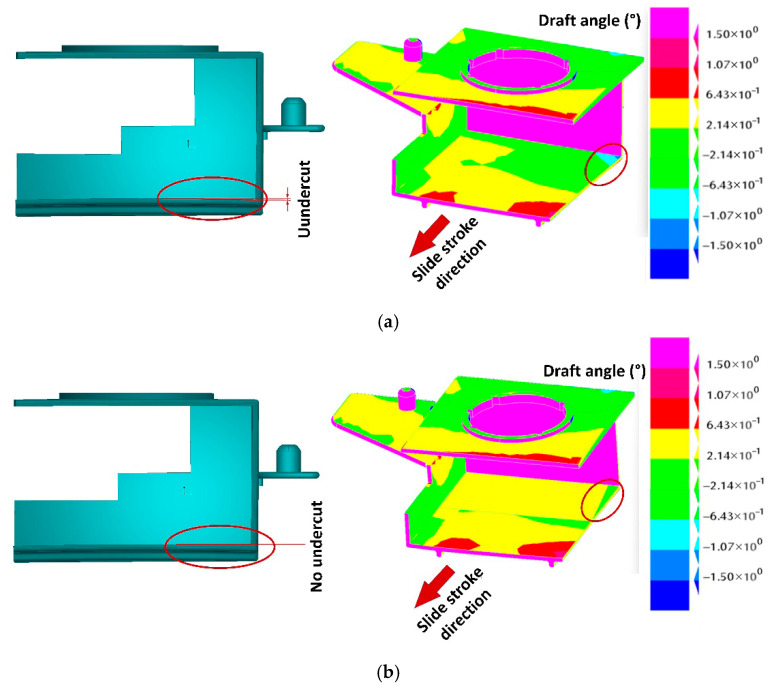
Redesigning molded part for moldabilty (red circles present the undercut area of the molded part): (**a**) warped geometry preventing slide from being pulled out from molded part: problematic area for ejection (left); result of surface analysis—darker green colored surfaces (with negative angle) inside the part represent undercuts in slide stroke direction (right). (**b**) Redesigned surfaces allowing smooth molded part ejection: view of redesigned surface (left), surface analysis of redesigned surface (right).

A CAD model of the molded part was used for an analysis of the moldability of such a deformed geometry. The most critical region in this case was the area formed by the mold slide. The surfaces aligned in the slide stroke direction must allow the slide to be pulled out of the part without any obstacles generated by the warped molded part geometry. An example of such an obstacle is shown in Figure 17a, while Figure 17b shows a redesigned solution.

#### 3.2.2. Inverse-Contoured Molded Part Injection Molding Simulation

**Figure 18:**
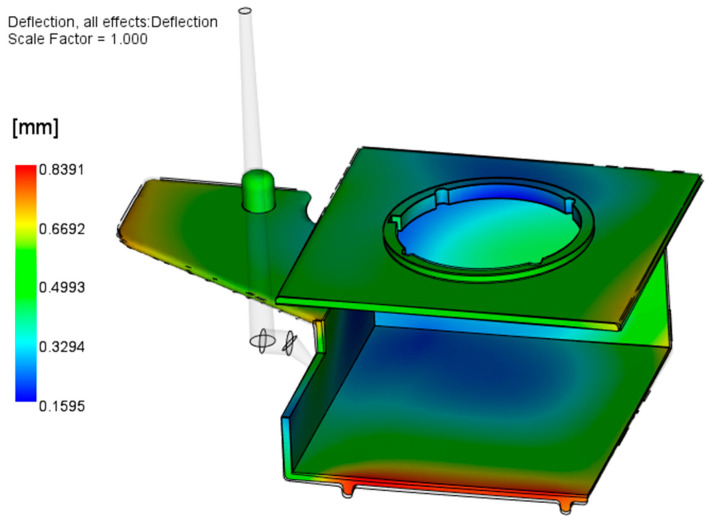
Results of Warp analysis with inverse-contoured molded part geometry.

In this step, the manually redesigned (inverse-contoured) warped molded part geometry was exported to Autodesk Moldflow Insight to carry out a final simulation process. The processing parameters for this simulation were the same as those obtained from the RSM optimization. The warpage analysis in Figure 18 shows the maximum warpage of 0.84 mm, but it should be considered that this deformation is in the opposite direction of the already deformed part.

#### 3.2.3. Warped Inverse-Contoured and Reference Molded Part Final Alignment and Validation

**Figure 19:**
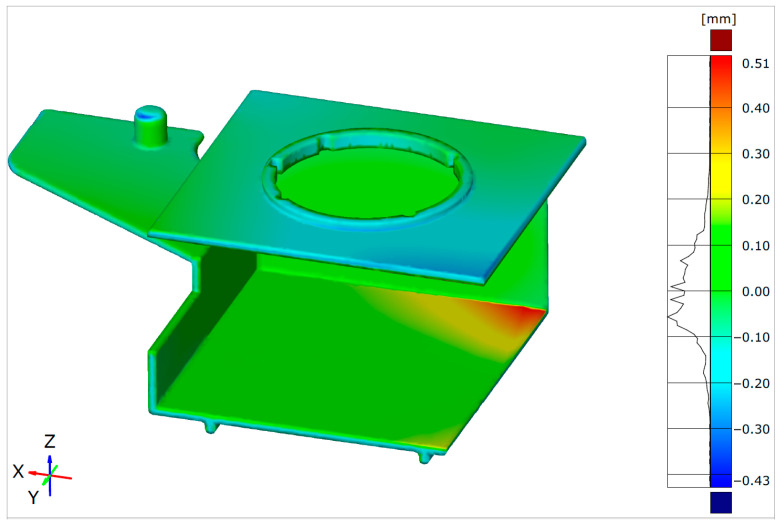
Surface Comparison after alignment—original CAD geometry with warped inverse-contoured geometry.

A final evaluation of the method was performed with the alignment of the warped inverse-contoured (actual) geometry with the reference CAD geometry of the molded part. Therefore, all the steps described in Section 3.1.3 were repeated. Figure 19 shows the results of the Surface Comparison of the warped inverse-contoured geometry with the molded part reference CAD geometry in ZEISS Inspect software.

**Figure 20:**
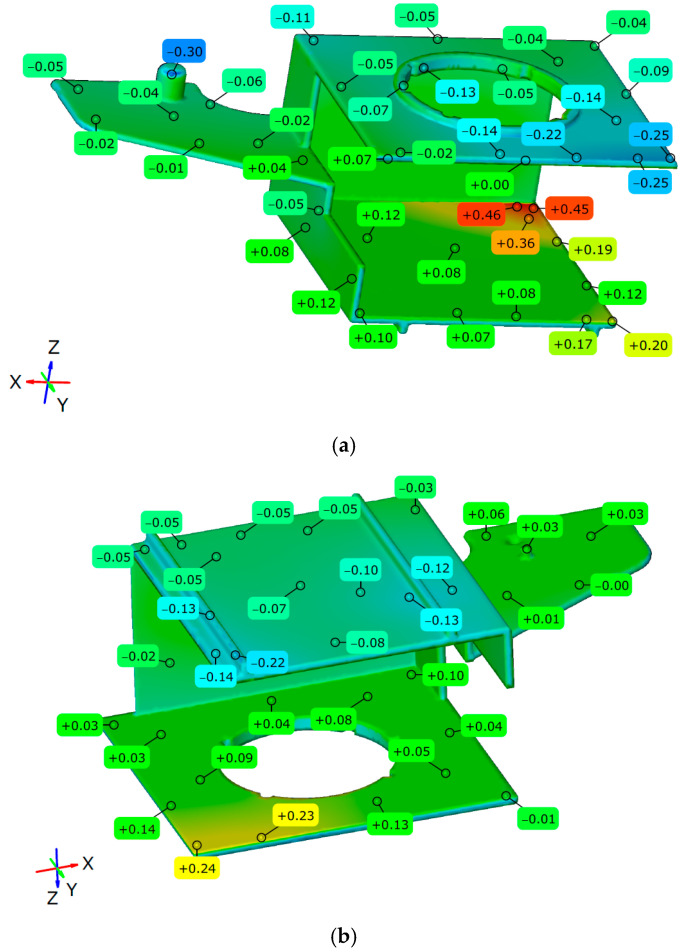
Surface deviation labels (deviations between reference CAD model and warped inverse-contoured part geometry): (**a**) upper view, (**b**) bottom view.

Figure 20 shows the results within the scale adjusted to show the maximum deviations in both directions with surface deviation labels.

By using the same scale as in the initial Surface Comparison, it is evident that the deviations from the nominal geometry are significantly reduced by approximately 82% (1.67 mm max with the original geometry vs. the majority of the surface deviations in the range ±0.30 mm with the inverse-contoured geometry). It is clear in this plot that a smaller deviation area with values around 0.50 mm is concentrated on the inner surface of the molded part, intentionally redesigned to allow the slide to be pulled from the molded part (as shown in Figure 17).

**Figure 21:**
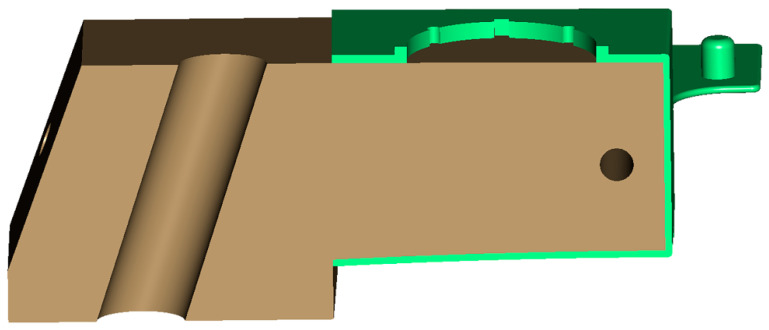
CAD model cross-section of the slide for shaping inverse-contoured molded part.

Based on the final inverse-contoured molded part geometry, the mold design process could start. Figure 21 shows a CAD model of a mold slide for an inverse-contoured molded part. It is obvious from the presented cross-section that there are no obstacles for the slide stroke from the molded part (undercuts in the stroke direction) during the mold opening phase.

## 4. Discussion

The inverse contouring method shown in this paper combines the application of injection molding computer simulation, RSM optimization, and the alignment and analysis of geometry deviations in inspection software. This method was applied in instances where the mold was already manufactured, which posed some obstacles in the optimization process (limited options for mold changes).

An injection molding simulation was initially used to determine the existing mold design and recommended injection molding parameters from Autodesk Moldflow Insight software and the polymer material technical datasheet. The result of the initial simulation showed significant warpage of the molded part, with a maximal value of 1.85 mm and a tendency to warp inward, leading to the conclusion that the mold slider was not efficiently cooled. When comparing two methods for improving slide cooling, the variation with an increasing slide cooling channel diameter was more efficient, allowing for a reduction in the maximal warpage of up to 1.06 mm. This is due to a more uniform temperature distribution within the mold cavity, which ensures more uniform cooling, reduces the level of residual stresses, and consequently reduces the warpage of the molded part. A uniform temperature distribution within the mold cavity was also found to be a key parameter for minimizing warpage in [22,33,66,67,68,69].

In the second step, Design Expert software was used to determine the DoE, namely, the CCD (RSM optimization), for a series of injection computer simulations aimed at warpage optimization via variations in melt, mold, and slide coolant temperatures. A statistical analysis confirmed that all three parameters are significant for molded part warpage, with the slide coolant temperature exerting the largest effect. The melt and mold temperatures have a moderate effect. These results are comparable with the results of other studies [38,53,67,68,69,71,74,77,78].

Based on these results, RSM optimization, with minimal warpage as the criterion, suggested the optimal injection molding parameters: a melt temperature of 280 °C, a mold temperature of 100 °C, and a slide inlet coolant temperature of 40 °C, with an estimated maximal molded part warpage of 0.57 mm. An injection molding simulation with the optimal parameters resulted in 0.73 mm, which is a higher value than the RSM optimization-estimated value but lower than the results obtained from all DoE runs. The reason for this deviation may be due to the fact that RSM optimization analyzed only three selected parameters and their interactions without taking into account the complex influences and interactions of other processing parameters affecting warpage. As the injection molding process is very complex, even changing the analysis method can significantly influence the results (an overview is shown in Table 1).

The Autodesk Moldflow Insight warpage simulation results with the RSM-optimized parameters (actual and opposite geometries) were exported for the next steps of the inverse contouring method.

The actual geometry was used for alignment and a more detailed analysis and prediction of the critical molded part dimensions/geometry deviations. The differences in the warpage levels shown in this paper were obvious. The initial warpage analysis results obtained via Autodesk Moldflow Insight were in the range from 0.19 to 1.85 mm, while the warped and reference part geometry deviation results obtained via ZEISS Inspect were in the range from −1.51 to 1.67 mm. The RSM-optimized Autodesk Moldflow Insight simulation results revealed a warpage range from 0.17 to 0.73 mm, while the ZEISS Inspect alignment results revealed a warpage from −0.76 to 0.72 mm.

The difference in the results arises from the fact that Autodesk Moldflow calculates the movement of FEM nodes of the warped geometry from their original positions in the x, y, and z directions and calculates the displacement of nodes in space (therefore, the values are always positive). ZEISS Inspect software, however, calculates the deviations of the warped geometry (points/surfaces) from the original geometry in vector form and both directions, positive and negative. Inspection software is aimed at precise measurements and quality control; therefore, it was presumed that alignment in this software can deliver more accurate results, which were used as the basis for further molded part analyses and inverse contouring.

Autodesk Moldflow Insight’s ability to generate opposite molded part geometries was utilized to generate the CAD geometry of the inverse-designed molded part. However, this does not take into account negative draft angles in the molded part or slide pull directions, which can prevent the molded part from being ejected. Therefore, the next step of the inverse contouring method involved an analysis and a manual redesign of the molded part to generate a geometry optimized for injection molding (mostly for ejection from the mold).

This geometry was used in the final step of inverse contouring for simulation and final alignment with the reference molded part geometry. The Autodesk Moldflow Insight simulation resulted in a maximal molded part warpage of 0.84 mm, but it was oriented in the opposite direction of the previously deformed geometry. The final result of the inverse contouring method was based on a comparison of the warped inverse-contoured actual geometry exported from Autodesk Moldflow Insight and the original (reference) molded part CAD geometry via alignment in ZEISS Inspect software. The final alignment showed that the inverse contouring method, combined with the previous simulation optimization (based on RSM), resulted in an approximately 82% warpage reduction compared to the initial simulation results without RSM optimization and inverse contouring (maximal warpage reduced from 1.67 mm to a range of ±0.30 mm according to the ZEISS Inspect results). Deviations in the inverse-contoured geometry exceeding ±0.30 mm originated from intentionally redesigned zones to allow slide pulling from the molded part. Table 7 presents a summary of the warpage results obtained at different steps of the inverse contouring method.

As already mentioned, warpage is highly affected by the polymer material and molded part geometry. Therefore, the results of warpage reduction in this study are somewhat comparable to the results presented in [98,99,100,101,102,103]. The methods in [98,99,100,101,102,103] were compared in [104] on five different molded part geometries. Shape optimization via the method presented in [99] resulted in warpage reduction in the range from 20 to 56%, while the method introduced in [100] resulted in warpage reduction in the range from 97 to 99%. The largest warpage reduction was recorded with the reverse design methods introduced in [101,103], with a warpage reduction of more than 99%.

The method presented in this paper is more practically oriented than those in the similar presented studies and utilizes available software for molded part warpage optimization. This method is complex and time-consuming, and it involves a combination of injection molding parameter optimization and molded part (and mold) inverse contouring. Practical implementation, therefore, still needs in-depth research, as advanced simulation capabilities and high computational resources may be necessary to successfully implement this method.

In this study, using only injection molding simulation combined with RSM, warpage was reduced by approximately 61%. Inverse contouring reduced warpage by an additional 21%, leading to a total of 82%. Therefore, in the case of high-value products, where dimensional and shape accuracy are high priorities, the inverse contouring method can result in a significant reduction in molded part warpage.

## 5. Conclusions

Obtaining accurate dimensional measurements is one of the biggest challenges in the production of polymer molded parts. Significant warping often causes molded parts to fail product validation or quality control. Warpage can be reduced by optimizing the mold runner system, tempering system, and injection molding parameters during mold design. As these optimization approaches result in moderate improvement (mainly up to 60%), methods based on optimizing the shape of the molded part in the opposite direction of warpage, i.e., inverse contouring, have been recently introduced.

In this paper, a method combining injection molding computer simulation, injection molding parameters, RSM optimization, alignment in specialized inspection software, and manual redesign is presented, demonstrating a reduction in warpage of approximately 82%.

Although this method includes a manual redesign, which can be seen as a drawback, this step is performed based on the simulated and aligned geometry, yielding satisfactory results and avoiding the typical trial-and-error approach in practice. The inverse contouring method, both as a sole method or combined with advanced optimization algorithms, is a promising method to reduce molded part warpage. Despite these advances, further improvements are needed to reduce computational demands and explore process control and variable optimization, enabling the practical implementation of these methods in an industrial setting.

Therefore, future research will focus on the evaluation of the inverse contouring method in a real injection molding process using a manufactured experimental mold.

## Figures and Tables

**Table 1:** Relevant research on optimization of significant injection molding parameters for minimizing warpage.

Literature	Method	Most Significant Effect on Warpage
Hopmann et al. [33]	simulation, experimental	mold temp. distribution, cooling rate
Lin et al. [9]	simulation, DoE, experimental	mold temp.
Kim et al. [22]	simulation, experimental	mold temp. distribution
Wang et al. [31]	simulation, experimental	packing time, packing pressure
Huang et al. [38]	simulation, experimental	injection time, mold temp. melt temp.
Hiyane-Nashiro et al. [46]	simulation, Taguchi, TOPSIS, GA	melt temp., injection time
Nitnara et al. [53]	simulation, ANN, GA	melt temp.
Lee et al. [56]	simulation	injection pressure, melt temp.
Sudsawibowo [65]	simulation, Taguchi, experimental	packing time, packing pressure
Kouchaki [66]	simulation, DoE	mold temp. distribution
Mohd et al. [67]	simulation, RSM, GA, experimental	coolant temperature (classic channels)
Oliaei et al. [68]	Taguchi, ANN	coolant temp., melt temp. cooling time (Taguchi: packing pressure)
Chen et al. [69]	simulation, Taguchi, ANN, experimental	melt temp., coolant temp., packing time
Huang et al. [70]	simulation, experimental	injection speed, pressure profile, V/P switchover
Kamarudin et al. [71]	Taguchi	mold temp., melt temp.
Hakimian et al. [72]	simulation, Taguchi	packing time, coolant temp.
Zheng et al. [73]	simulation, Taguchi	packing pressure, melt temp., packing time
Chen et al. [74]	simulation, Taguchi, experimental	ambient temp., melt temp., mold temp.
Sudsawat et al. [75]	simulation, RSM	packing time, cooling time, melt temp.
Farshi et al. [76]	simulation, simplex algorithm	cycle time
Kitayama et al. [77]	simulation, sequential optimization	mold temp., melt temp.
Kang et al. [78]	simulation, adaptive optimization method	melt temp., mold temp., packing pressure, packing time
Zhang et al. [79]	simulation	cooling channels designs, packing pressure, packing time, cooling time, mold temp., melt temp.
Sandu et al. [80]	DoE, experimental	packing pressure, melt temp. injection speed

DoE—design of experiment, TOPSIS—Technique for Order of Preference by Similarity to Ideal Solution. GA—Generic Algorithm, ANN—Artificial Neural Network, V/P—velocity/pressure.

**Table 2:** PBT-GF30 material—relevant properties [105].

Property	Standard	Unit	Value
**Rheological**			
Melt flow rate	ISO 1133-1	cm^3^/(10 min)	17
Shrinkage—parallel	ISO 294-4	%	0.4
Shrinkage—perpendicular	ISO 294-4	%	1.1
**Mechanical**			
Tensile modulus	ISO 527-1,-2	MPa	9800
Tensile stress at break	ISO 527-1,-2	MPa	140
Flexural modulus	ISO 178-A	MPa	9800
Flexural strength	ISO 178-A	MPa	225
Charpy impact strength (23 °C)	ISO 179-1eU	kJ/m^2^	65
**Thermal**			
Melt temperature	ISO 11357-1,-3	°C	225
Thermal conductivity	ISO 8302	W/(m·K)	0.26
**Processing**			
Melt temperature (molding)	ISO 294		250–270
Mold temperature (molding)	ISO 294		80–100

**Table 3:** Main boundary condition data [106].

Type of Boundary Condition	Unit	Value
Molded part mesh	tetrahedral elements	832,185
Cooling system mesh	beam elements	36
Cooling circuit inlet/outlet	number of inlets/outlets	6
Mold block mesh	tetrahedral elements	632
Melt temperature	°C	260
Mold temperature	°C	80
Coolant temperature	°C	60
Part ejection temperature	°C	187

**Table 4:** Central composite design for warpage optimization (15 runs).

Run Number	Melt Temp. (°C)	Target Mold Temp. (°C)	Coolant Inlet Temp. (°C)	Max. Warpage (mm)
1	250	80	60	1.094
2	260	107	70	1.332
3	270	100	80	1.499
4	270	100	60	0.991
5	260	90	80	1.544
6	260	90	87	1.726
7	260	90	70	1.285
8	270	80	60	1.041
9	250	100	60	1.116
10	243	90	70	1.352
11	277	90	70	1.210
12	250	80	70	1.301
13	250	100	80	1.651
14	260	73	70	1.243
15	270	100	53	0.872

**Table 5:** ANOVA of warpage (quadratic model, DoF—degree of freedom).

Source	Sum of Squares	DoF	Mean Square	F-Value	*p*-Value	Remark
Model	0.8386	9	0.0932	536.55	<0.0001	significant
**A—melt temp.**	0.0245	1	0.0245	141.30	<0.0001	significant
**B—mold temp.**	0.0050	1	0.0050	28.79	0.0030	significant
**C—coolant temp.**	0.3874	1	0.3874	2230.70	<0.0001	significant
**AB**	0.0020	1	0.0020	11.28	0.0201	significant
AC	0.0003	1	0.0003	1.88	0.2282	
**BC**	0.0024	1	0.0024	13.95	0.0135	significant
A^2^	0.0001	1	0.0001	0.4811	0.5188	
B^2^	0.0000	1	0.0000	0.1181	0.7450	
**C^2^**	0.0019	1	0.0019	10.85	0.0216	significant
Residual	0.0009	5	0.0002			
Corrected total	0.8395	14				

*p*-values less than 0.0500 indicate that the model terms are significant. In this case, A (melt temperature), B (mold temperature), and C (coolant temperature), as well as interactions AB, BC, and C^2^, are significant model terms.

**Table 6:** RSM optimization criteria.

Parameter	Goal	Lower Limit	Upper Limit	Level of Importance
A—melt temp.	In range	240 °C	280 °C	3
B—mold temp.	In range	70 °C	110 °C	3
C—coolant temp.	In range	40 °C	80 °C	3
**Warpage**	**Minimize**	**0.50**	**0.90**	**5**

**Table 7:** Summary of molded part warpage results.

Inverse Contouring Step	Software	Warp. Max. Value (mm)	Warp. Decreasing (%) *
Initial simulation	Moldflow	1.85	-
Initial simulation (increased cooling channel diam.)	Moldflow	1.06	43
RSM optimization	Design Expert	0.57	71
RSM optimization	Moldflow	0.73	61
Inverse-contoured **	Moldflow	0.84	-
Initial simulation	ZEISS Inspect	−1.51 +1.67	-
RSM optimization	ZEISS Inspect	−0.76 +0.72	(−)50 (+)57
Inverse-contoured ***	ZEISS Inspect	±0.30	~82

* Decreasing % is based on the initial result, ** warpage in opposite direction, *** majority of deviations within the range.

## Data Availability

Data are contained within the article.

## Supplementary Materials

The following supporting information can be downloaded at: https://www.mdpi.com/article/10.3390/polym17172278/s1, Figure S1: Main dimensions of molded part, Figure S2: Mold sketch—movable mold plate, Table S1. Injection molding simulation runs—warpage results.

## Abbreviations

The following abbreviations are used in this manuscript:

PBT	Polybutylene terephthalate
STL	Standard tessellation language
CAD	Computer-Aided Design
DSC	Differential scanning calorimetry
V/P	Velocity/pressure
DoE	Design of experiment
TOPSIS	Technique for Order of Preference by Similarity to Ideal Solution
GA	Generic Algorithm
ANN	Artificial Neural Network
ANOVA	Analysis of variance
S/N	Signal-to-noise ratio
CCC	Conformal cooling channel
FEM	Finite element method
GF	Glass fiber
CCD	Central composite design
DoF	Degree of freedom
RE	Reverse engineering
