# Supplementary material for: Molded Part Warpage Optimization Using Inverse Contouring Method
Source: Polymers (Basel). 2025 Aug 22;17(17):2278. doi: 10.3390/polym17172278 (PMC12431507; doi:10.3390/polym17172278)
Supplement: Supplementary file 1 [file polymers-17-02278-s001.zip › polymers-3783176-supplementary.pdf]

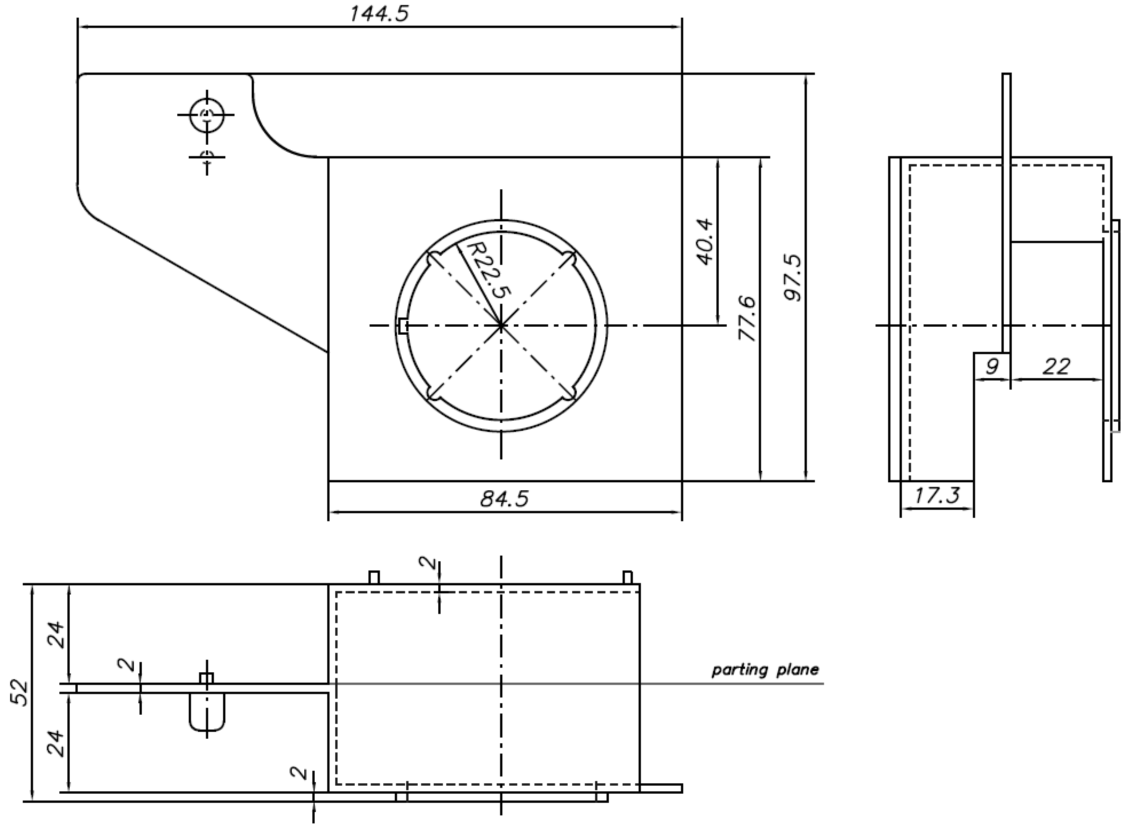

Figure S1: Main dimensions of molded part.

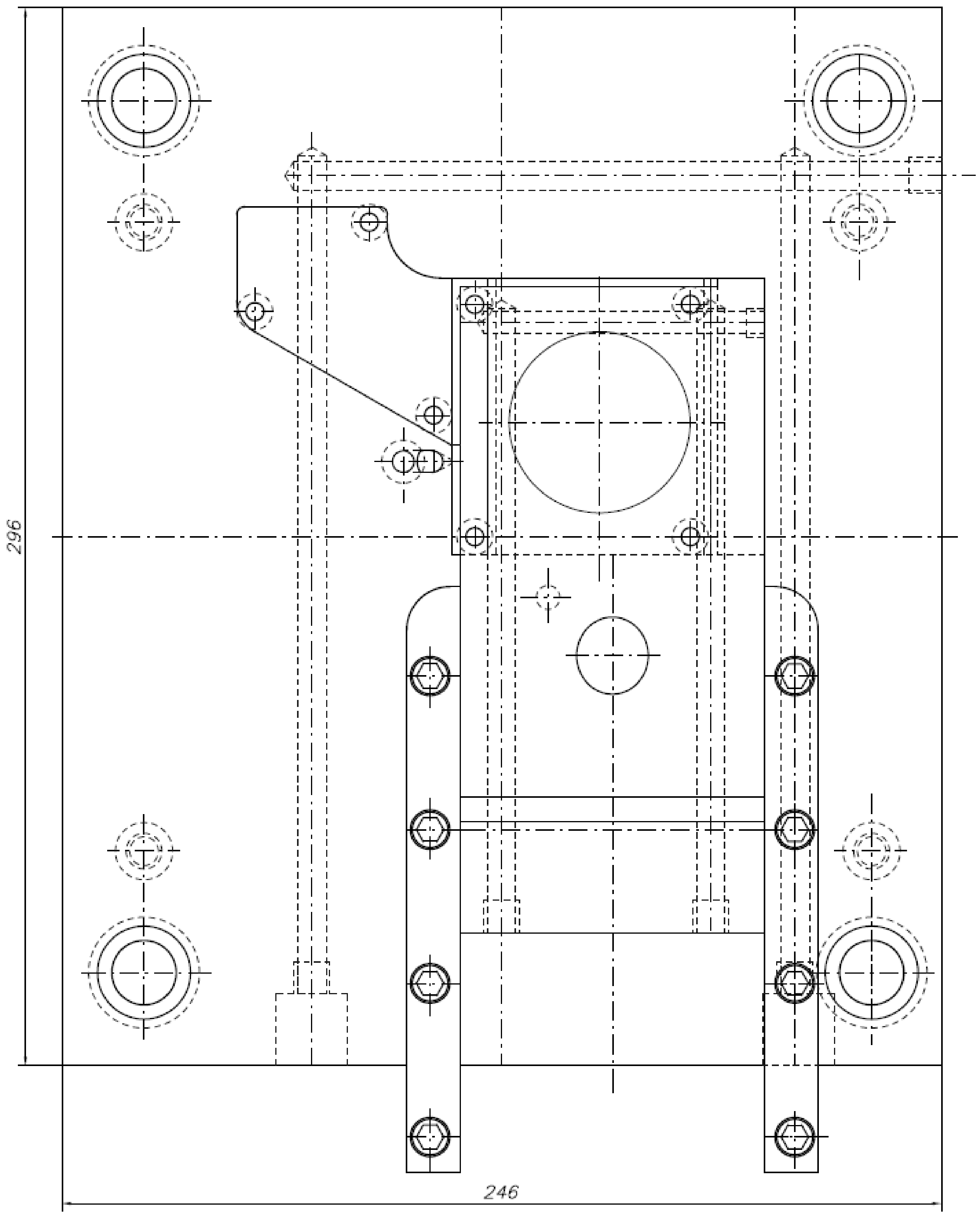

Figure S2: Mold sketch – movable mold plate.

Table S1. Injection molding simulation runs – warpage results.

Run	Variable parameter	Warpage (deformation visualization scale – 2x)
1	Melt temp.: 250 °C<br>Mold temp.: 80 °C<br>Coolant temp.: 60 °C	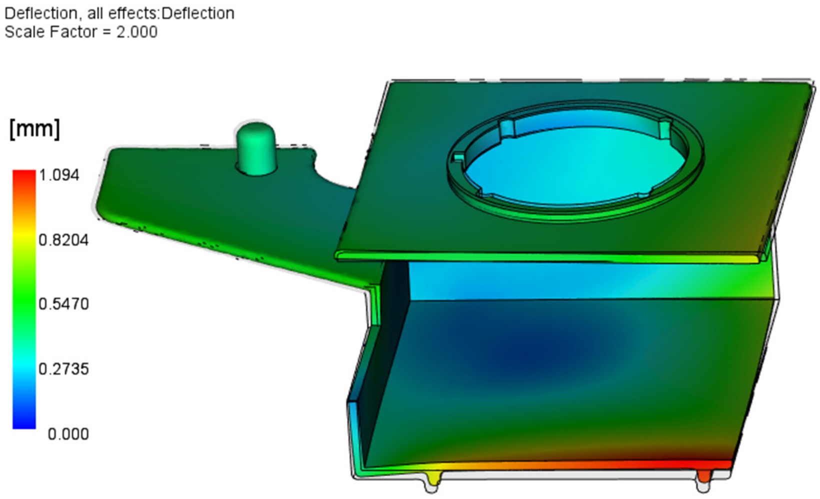
2	Melt temp.: 260 °C<br>Mold temp.: 107 °C<br>Coolant temp.: 70 °C	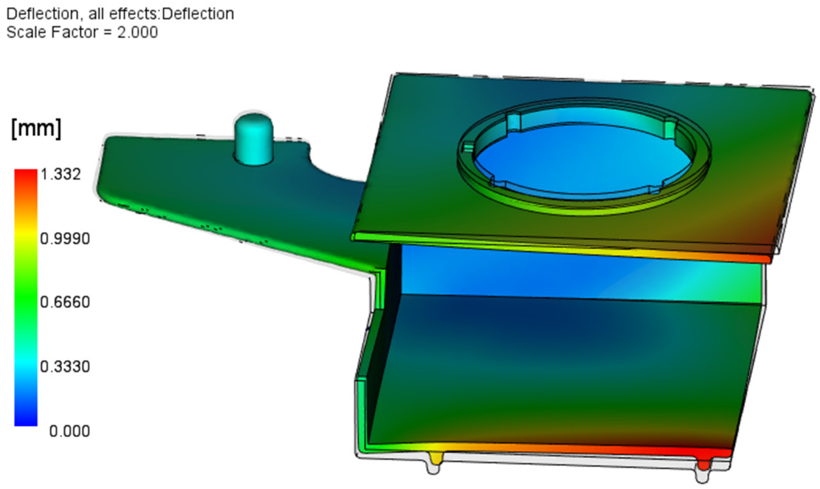
3	Melt temp.: 270 °C<br>Mold temp.: 100 °C<br>Coolant temp.: 80 °C	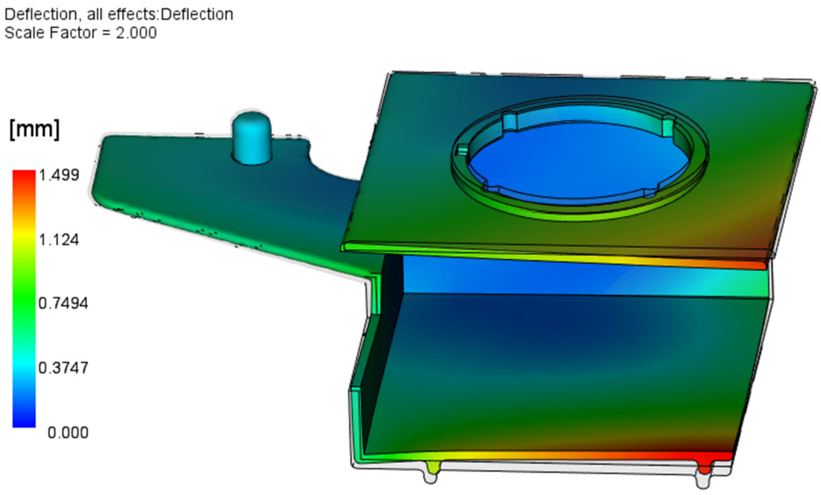

4	Melt temp.: 270 °C<br>Mold temp.: 100 °C<br>Coolant temp.: 60 °C	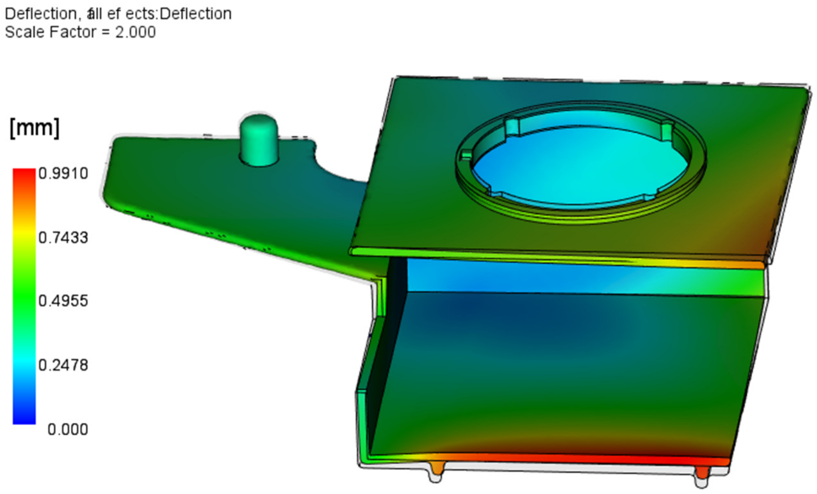
5	Melt temp.: 260 °C<br>Mold temp.: 90 °C<br>Coolant temp.: 80 °C	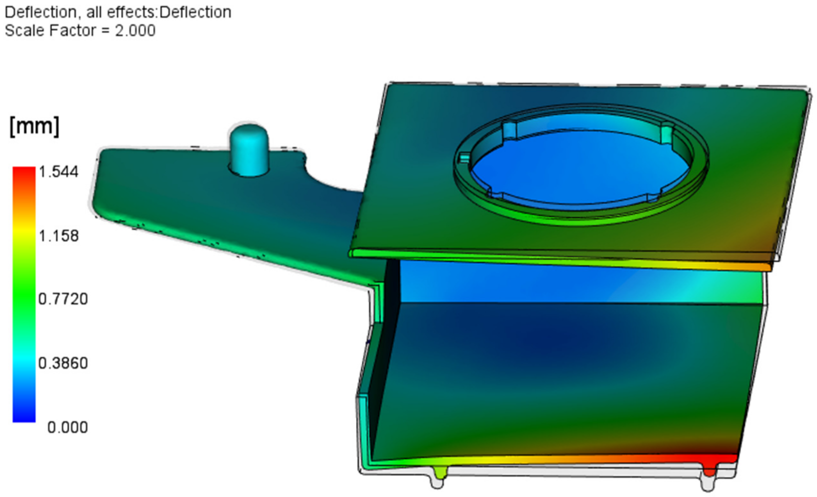
6	Melt temp.: 260 °C<br>Mold temp.: 90 °C<br>Coolant temp.: 87 °C	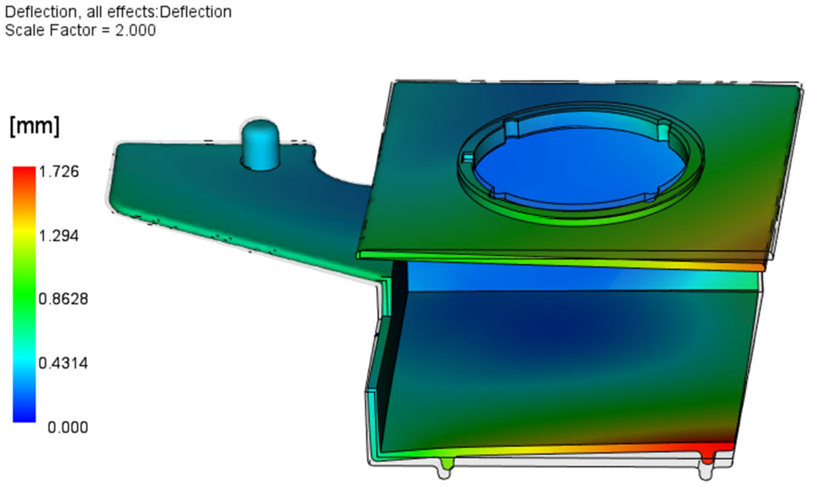

7

Melt temp.: 260 °C
Mold temp.: 90 °C
Coolant temp.: 70 °C

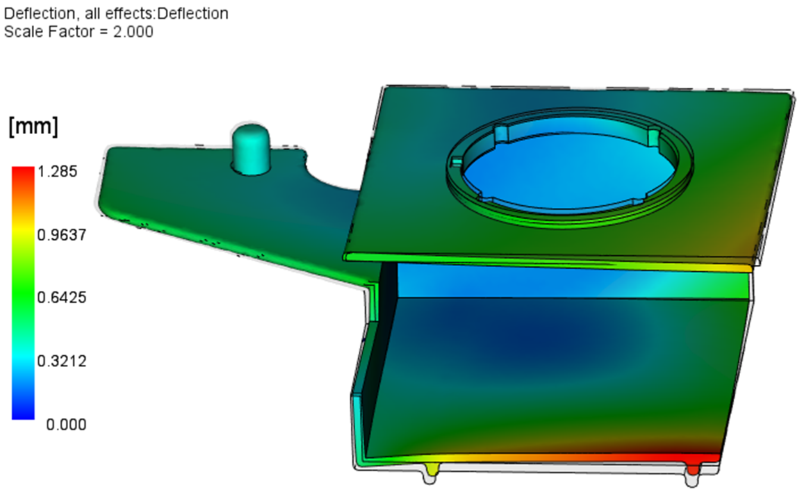

8

Melt temp.: 270 °C
Mold temp.: 80 °C
Coolant temp.: 60 °C

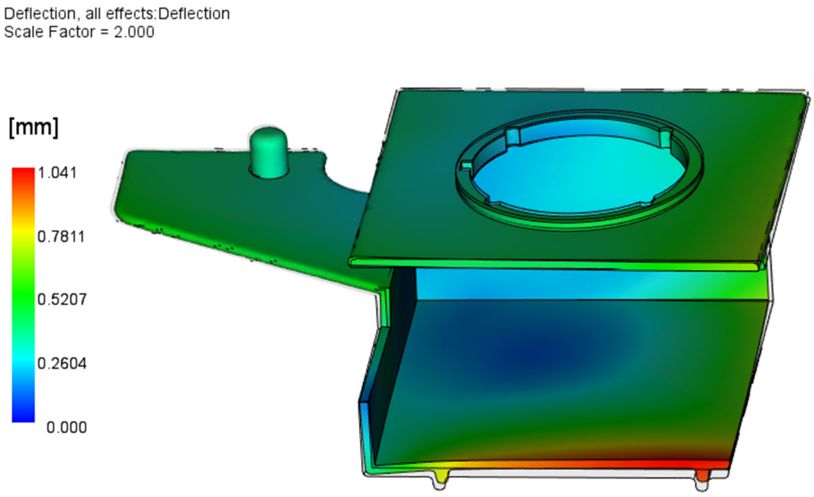

9

Melt temp.: 250 °C
Mold temp.: 100 °C
Coolant temp.: 60 °C

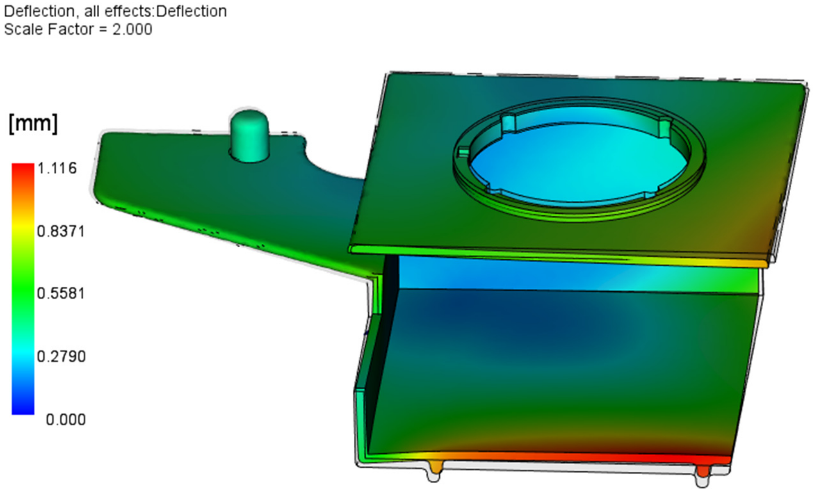

10	Melt temp.: 243 °C<br>Mold temp.: 90 °C<br>Coolant temp.: 70 °C	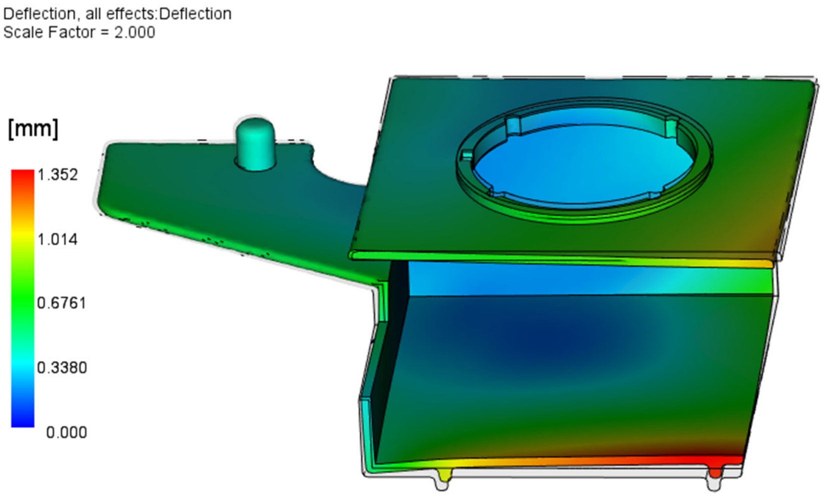
11	Melt temp.: 277 °C<br>Mold temp.: 90 °C<br>Coolant temp.: 70 °C	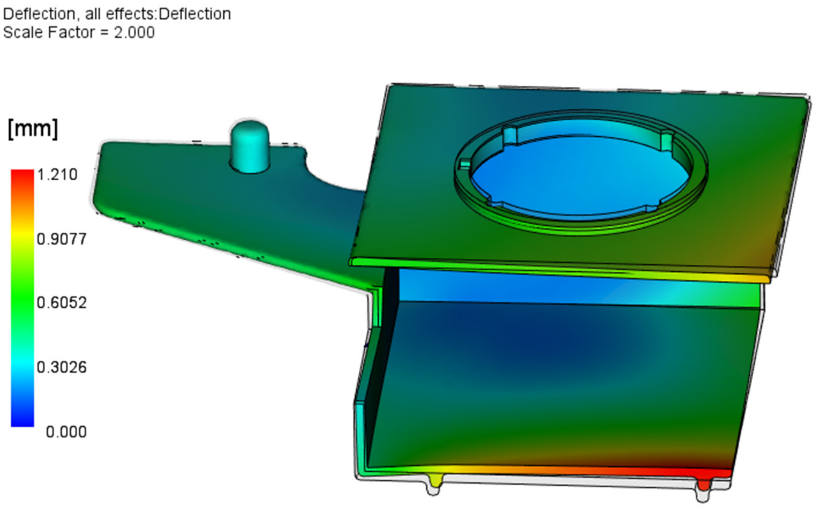
12	Melt temp.: 250 °C<br>Mold temp.: 80 °C<br>Coolant temp.: 70 °C	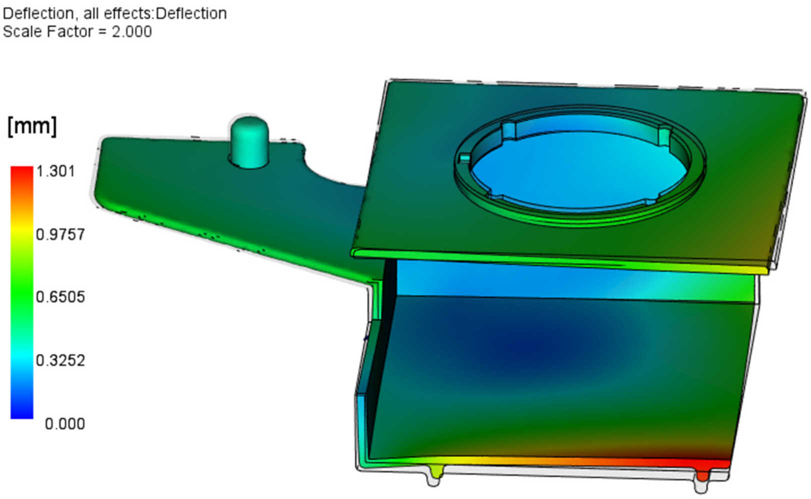

13	Melt temp.: 250 °C<br>Mold temp.: 100 °C<br>Coolant temp.: 80 °C	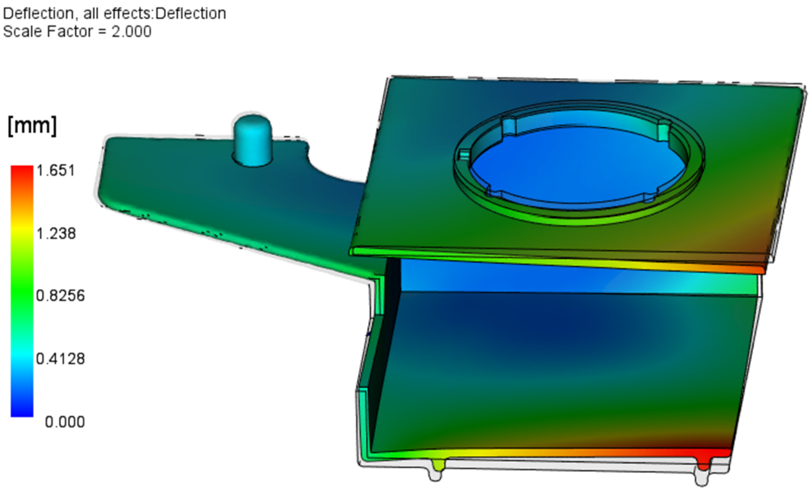
14	Melt temp.: 260 °C<br>Mold temp.: 73 °C<br>Coolant temp.: 70 °C	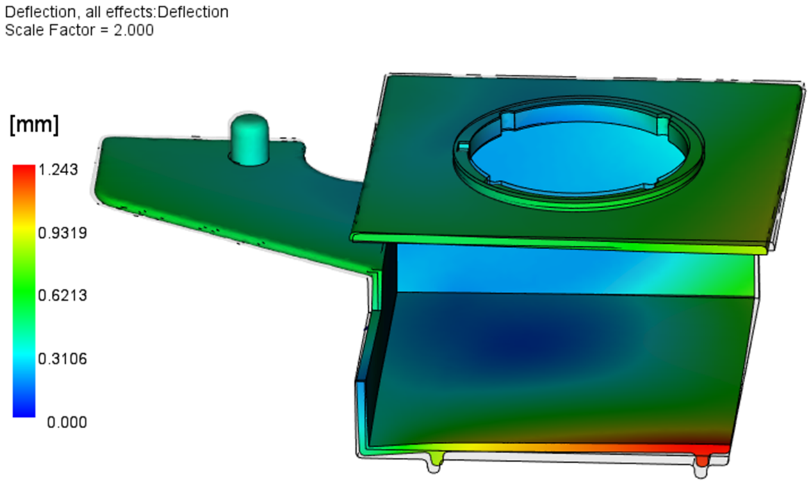
15	Melt temp.: 270 °C<br>Mold temp.: 100 °C<br>Coolant temp.: 53 °C	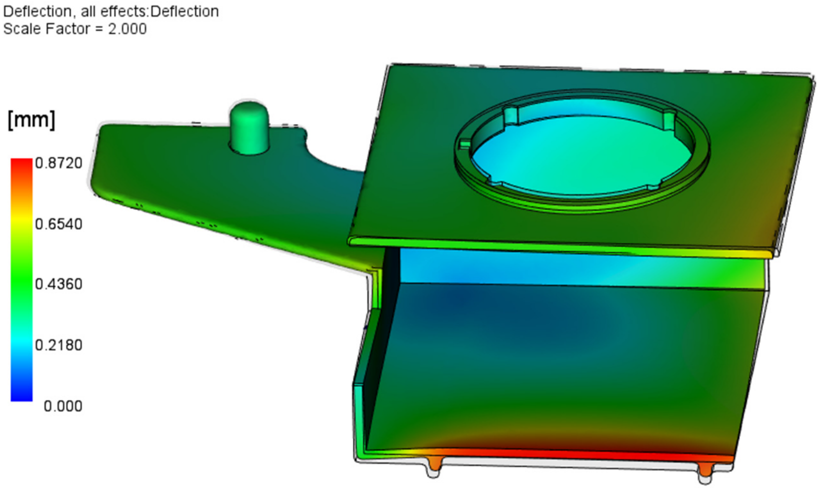
